# Naturally Occurring Norsteroids and Their Design and Pharmaceutical Application

**DOI:** 10.3390/biomedicines12051021

**Published:** 2024-05-06

**Authors:** Valery M. Dembitsky

**Affiliations:** Centre for Applied Research, Innovation and Entrepreneurship, Lethbridge College, 3000 College Drive South, Lethbridge, AB T1K 1L6, Canada; valery.dembitsky@lethbridgecollege.ca or devalery@gmail.com

**Keywords:** norsteroids, meroterpenoids, invertebrates, seaweeds, fungal endophytes, fungi, plant, activity

## Abstract

The main focus of this review is to introduce readers to the fascinating class of lipid molecules known as norsteroids, exploring their distribution across various biotopes and their biological activities. The review provides an in-depth analysis of various modified steroids, including A, B, C, and D-norsteroids, each characterized by distinct structural alterations. These modifications, which range from the removal of specific methyl groups to changes in the steroid core, result in unique molecular architectures that significantly impact their biological activity and therapeutic potential. The discussion on A, B, C, and D-norsteroids sheds light on their unique configurations and how these structural modifications influence their pharmacological properties. The review also presents examples from natural sources that produce a diverse array of steroids with distinct structures, including the aforementioned A, B, C, and D-nor variants. These compounds are sourced from marine organisms like sponges, soft corals, and starfish, as well as terrestrial entities such as plants, fungi, and bacteria. The exploration of these steroids encompasses their biosynthesis, ecological significance, and potential medical applications, highlighting a crucial area of interest in pharmacology and natural product chemistry. The review emphasizes the importance of researching these steroids for drug development, particularly in addressing diseases where conventional medications are inadequate or for conditions lacking sufficient therapeutic options. Examples of norsteroid synthesis are provided to illustrate the practical applications of this research.

## 1. Introduction

Steroids are de facto isoprenoid lipids and are found in eukaryotic organisms, from microorganisms to macroalgae, invertebrates, and plants, and are present in most sedimentary organic matter [1,2,3,4,5,6,7,8]. Thus, steroids and isoprenoid lipids are an important group of fossil chemical compounds that provide valuable information about the sources of organic matter in modern sediments and ancient sedimentary rocks, as well as crude oil [9,10,11,12]. Diagenesis of organic matter found in waters and sediments modifies the structures of steroid precursors in complex ways.

The concept of “norsteroids” was introduced in the scientific literature by American chemist Russell Marker in the early 1930s. Marker discovered a way to synthesize hormones such as progesterone from plant sterols, leading to the development of the first oral contraceptive pill. He coined the term “norsteroids” to describe these compounds, which are structurally related to steroids but have a carbon removed from the steroid nucleus. This term has since been used to refer to a class of compounds that are derived from steroids but have modified structures [13]. Marker was a pioneering figure in the field of steroid chemistry and made significant contributions to the synthesis of various steroid compounds, including the development of methods to produce progesterone from diosgenin, a compound found in plants. His work laid the foundation for the production of cortisone and other important steroid medications [14,15].

**Figure 1:**
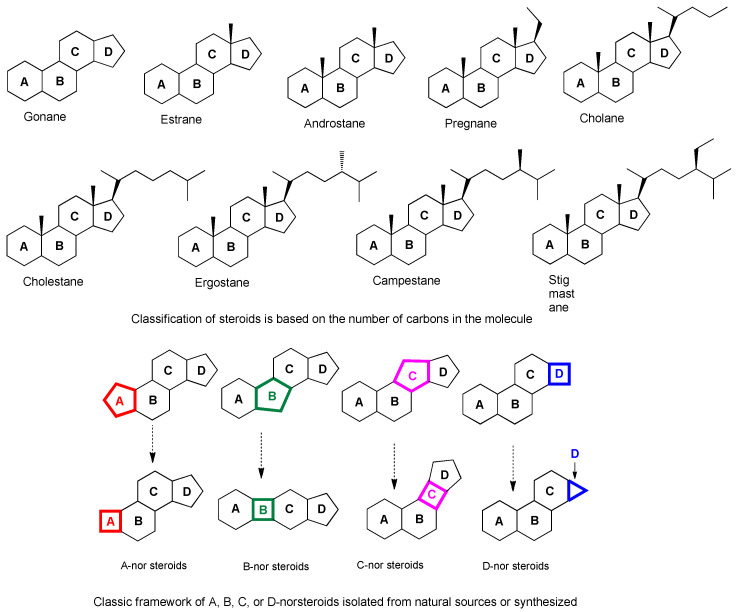
Steroids are categorized into various groups based on their carbon content. Gonanes, comprising 17 carbons, are one such group. Estranes, another group, consist of 18 carbons (C18 steroids) and include estrogens. Androstanes, encompassing 19 carbons (C19 steroids), are known for including androgens. Pregnanes, contains 21 carbons (C21 steroids), include important hormones like progesterone and corticosteroids. Cholanes, with 24 carbons, are associated with bile acids. Cholestanes, which have 27 carbons, include cholesterol and similar compounds. Ergostane, a tetracyclic triterpene, also known as 24S-methylcholestane, and campestane or 24R-methylcholestane are tetracyclic triterpenes (both have 28 carbon atoms). Lastly, stigmastane or 24R-ethylcholestane is a tetracyclic triterpene and has 29 carbon atoms. Structurally, steroidal hormones are characterized by three cyclohexane rings (labeled A, B, and C) and one cyclopentane ring (D), connected in a specific manner. Alterations in the structure of these rings lead to the formation of norsteroids.

Norsteroids (see Figure 1) are natural and/or synthetic isoprenoid lipids that have undergone any ring size reduction (removal of one carbon atom) or side chain reduction (removal of methyl groups) by biosynthetic or synthetic means. 

Based on these de facto principles, four groups of steroids or triterpenoids can be distinguished [1,2,3,4,5,7,14,15]. The first group includes steroids in which the A ring is reduced by one carbon atom. These are A-norsteroids and they are natural or synthetic isoprenoid lipids that have undergone any ring size reduction (removal of one carbon atom) or side chain reduction (removal of methyl groups) by biosynthetic or synthetic means. The second group includes steroids in which the B ring is reduced by one carbon atom (or two carbons). The third group includes steroids in which the C ring is reduced by one carbon atom. These are C-norsteroids. The fourth group includes steroids in which the D ring is reduced by one or two carbon atoms, and these are D-norsteroids [16,17]. The most interesting for this review is the information about A, B, C, and D-norsteroids, which are found in marine organisms and algae and are also synthesized by fungi or fungal endophytes [1,2,3,4,5,6,7,8,16,17].

This review presents information on the content of these steroids in various biotopes, as well as the remains of these compounds found in marine and freshwater sediments and sedimentary rocks. Also of interest is information on the biological activity of A, B, C, and D-norsteroids, which is demonstrated by both natural and synthetic analogs.

## 2. A-Norsteroids and Triterpenoids Derived from Marine Sources

A-norsteroids, also known as norsteroids or A-nor analogs, are a class of organic compounds that are structurally derived from steroids by removing one carbon atom from the steroid framework. Specifically, the carbon atom that is removed is located between the A and B rings of the steroid structure, leading to the prefix “A-nor” (meaning “without A”) in the name. The removal of this carbon atom results in an open or missing ring structure in the A ring of the steroid molecule. This modification alters the overall shape and properties of the steroid, leading to changes in its biological activity and pharmacological properties [18,19,20,21,22,23].

A-norsteroids can be synthesized through various chemical methods, including rearrangements, oxidations, or reductions of existing steroids. They have been of particular interest in medicinal chemistry and drug development due to their potential to exhibit unique biological activities and improved therapeutic profiles compared to the parent steroids. These compounds have been investigated in various therapeutic areas, such as anti-inflammatory, anticancer, and hormonal therapies. Some examples of A-norsteroids include norethisterone (a synthetic progestin), nor-androstenedione (a precursor to the anabolic steroid nandrolone), and norbolethone (a synthetic anabolic steroid) [20,21,22,23].

It is important to note that the specific properties and effects of A-norsteroids can vary depending on the exact structural modifications made and the specific compound under consideration. Therefore, it is necessary to evaluate each A-norsteroid individually in terms of its structure, biological activity, and potential applications.

**Figure 2:**
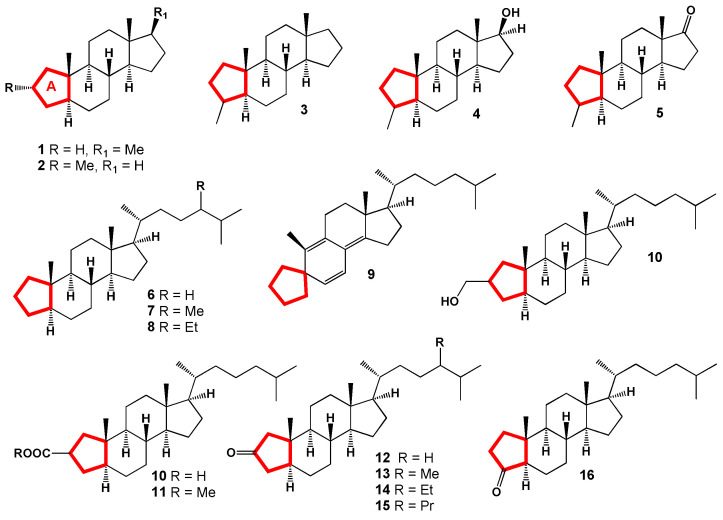
A-norsteroids derived from sediments, crude oils, and fossil organic biomarkers.

**Figure 3:**
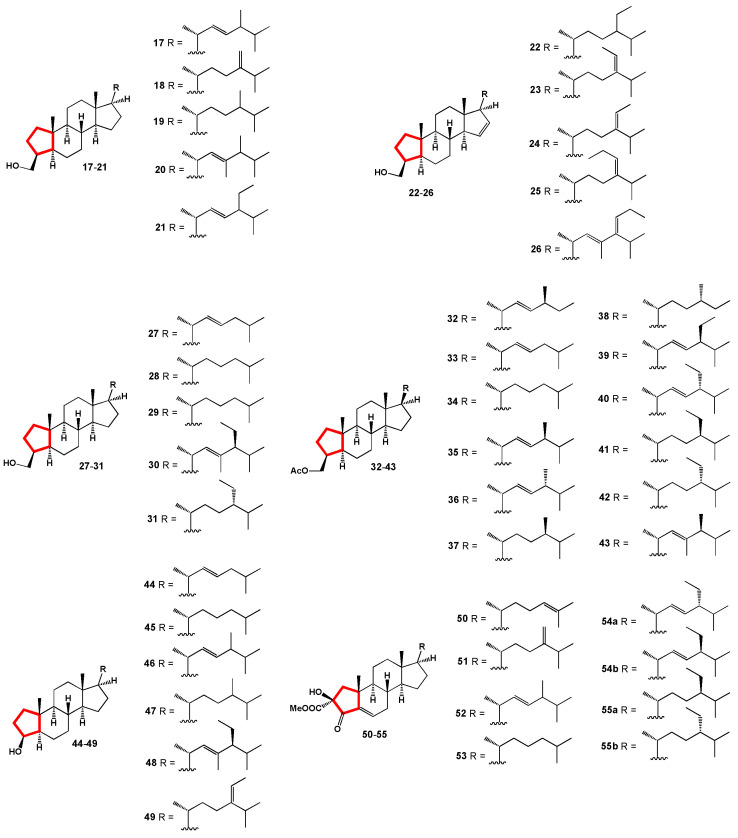
A-norsteroids derived from marine species.

**Figure 4:**
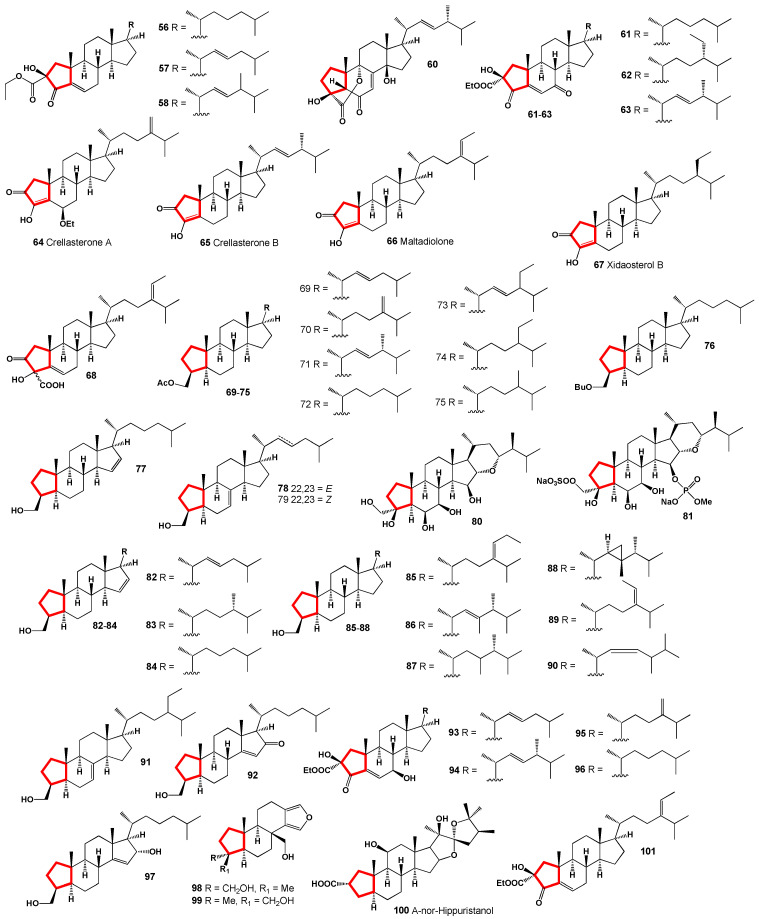
A-norsteroids identified from marine invertebrates and seaweeds.

**Figure 5:**
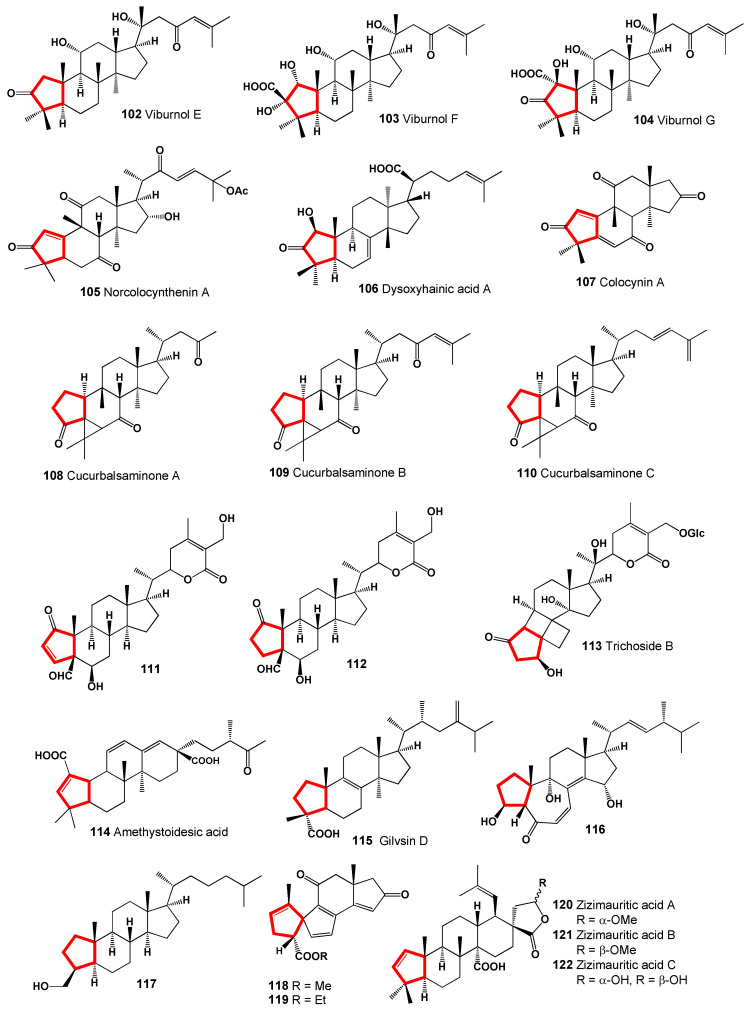
A-norsteroids isolated from plant extracts or individual parts thereof.

**Figure 6:**
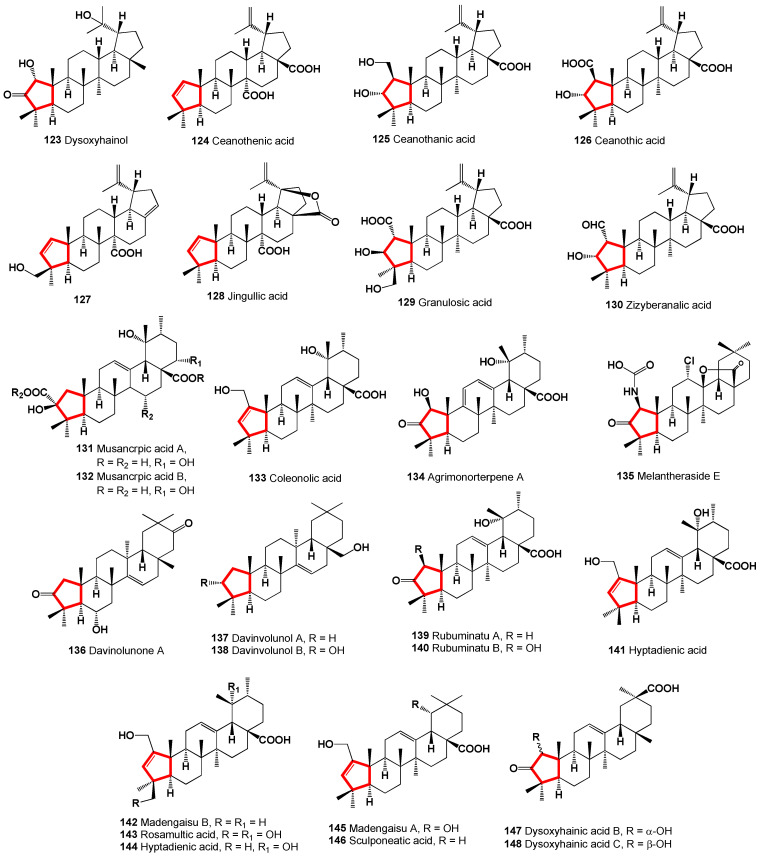
A-norsteroids isolated from extracts of plants or their individual parts, bark, leaves, fruits.

The study of ancient sediments, crude oils, and marine and freshwater sediments for their steroid and other isoprenoid lipid contents is crucial for both academic research and practical applications [6,9,24,25,26]. This area of research provides insights into the biological and chemical transformations of lipid molecules over extended periods under varying temperature and geochemical conditions. Bacteria play a pivotal role in the mineralization of steroids in the biosphere. The aerobic breakdown of steroid hormones relies on oxygen as a co-substrate for oxygenases, which activate and cleave the resilient steroid core ring. In anaerobic environments, denitrifying bacteria utilize pathways to decompose various steroid structures. Recent metaomics studies reveal that microorganisms can alter or convert steroid molecules into a wide array of intriguing compounds found across diverse ecosystems. Among the numerous steroids identified, a small subset known as norsteroids [6,9,24,25,26,27,28] is particularly notable. Figure 2 illustrates most common A-norsteroids (**1–16**) which are found in crude oil extracts, marine sediments, and other geological and environmental sources. The deformed A ring in A-norsteroids (**1–188**) is highlighted in red in Figure 2, Figure 3, Figure 4, Figure 5 and Figure 6. 

Sponges, belonging to the Porifera phylum, are some of the earliest multicellular organisms, with fossils dating back over 580 million years to the Precambrian era. Sponges play a significant role in tropical reefs, often dominating the biomass, and are also present in polar and deep ocean environments, as well as freshwater habitats. Both marine and freshwater sponges are sources of various biologically active compounds, including A-norsteroids [1,2,3,4,5,6,29,30].

Although sponges are recognized as sources of bioactive substances, the exact origin of these compounds remains unclear. Sponges are known to form symbiotic relationships with bacteria, fungal endophytes, and microalgae [31,32]. Despite this, the metabolites isolated from sponges are categorized as their own compounds. A-norsteroids derived from sponges are detailed below.

The marine sponge *Axinella carteri*, harvested from the Bay of Bengal off the Orissa coast, was found to contain A-nor 5α-cholestan-2-one (**12**) [33]. This particular steroid has been identified as an agonist for nerve growth factor [34]. In Western Australia’s ocean waters, sponges such as *Agelas mauritiana*, *Clathria major*, *Darwinella australiensis*, *Didiscus aceratus*, *Haliclona* sp., and *Teichaxinella labirintica* have been collected and analyzed for their steroid content [35]. From these sponges, approximately 60 different steroids were isolated, with A-norsteroids (**17–26**) among them. Notably, extracts from *Agelas mauritiana* and *Darwinella australiensis* have displayed hemolytic activities [33,34,35,36].

A collaborative team of French and Saudi Arabian scientists investigated various sponges along the Senegalese coast, including *Pseudosuberites* sp., *Suberites massa*, *Suberites* sp.1, *Suberites* sp.2, and *Rhizaxinella elongata*. Additionally, three *Ciocalypta* species (sp.1, sp.2, sp.3) were collected from Dakar, and *Stylissa carteri* was gathered from the Red Sea near Jeddah, Saudi Arabia [37]. Analysis of these sponges revealed the presence of eight A-norsteroids (**19**, **27–31**, structures in Figure 3) in their extracts. Furthermore, the steroid 3-hydroxymethyl-24-methyl-A-norcholestane (**19**) was identified in *Axinella verrucosa*, *Hymeniacidon perlevis*, *Homaxinella trachys*, *Phakellia aruensis*, and the triton *Charonia tritonis* [38,39,40]. The compound 24-ethyl-3-hydroxymethyl-A-norcholestane (**31**) was detected in *Acanthella aurantiaca*, *Axinella verrucosa*, and *Homaxinella trachys* [38,40]. A-Norcholistane-3-methanol (**28**) was found in *Axinella verrucosa* and *Hymeniacidon aldis* [41,42,43,44], and (3β,5α,22*E*,24*R*)-3-Hydroxymethyl-24-methyl-A-norcholest-22-ene (**36**) was isolated from these same sponges [41,42,43,44].

Researchers from the University of California focused on the Gulf of Mexico sponge *Teichaxinella morchella*, discovering that its extracts contained exclusively 3-hydroxymethyl-A-nor-sterols (**32–43**). The exclusive presence of A-norsterols, with no normal sterols, indicates that these compounds might be biotransformation products of dietary precursors or result from bacteria transforming common sterols into A-norsterols [41,42,45].

Bulgarian lipidologists studied *Halichondria panicea* and *Hymeniacidon sanguinea* from the Black Sea, finding A-norsterols in both sponges (**44–49**). The similarity in sterol composition led scientists to hypothesize a shared diet or other food sources [46].

The Florida sponge *Phorbas amaranthus* is known to contain A-norsteroids, specifically anthosterones A (**50**) and B (**51**), and other steroids named phorbasterones A–D (**52–55**). These compounds exhibited moderate cytotoxicity against HCT-116 tumor cells [47]. Anthosterones A (**50**) and B (**51**) were previously isolated from the sponge *Anthoarcuata graceae* in British Columbia’s Deer Group Islands [48]. The red alga *Calloseris* sp. (family Delesseriaceae), found along Madagascar’s eastern coastline, yielded phorbasterone B (**53**), also found in *Phorbas amaranthus*, and another A-norsteroid, (−)-2-ethoxycarbonyl-2β-hydroxy-A-norcholest-5-en-4-one (**56**) [49]. This steroid (**56**, see the structure in Figure 3) was also detected in the soft coral *Acropora formosa* [50]. *Lasiodiplodia pseudotheobromae*, a pathogenic fungus from the Botryosphaeriaceae family, produced two steroids: phorbasterone A (**52**) and the unusual 3β,14β-dihydroxy-6-oxo-A-nor-ergosta-7,22-diene-4-oic acid δ-lactone (**60**) [51]. This fungus, found on Chinese carcasses and *Acacia mangium* in Venezuela, causes mango fruit rot. Additionally, the steroid phorbasterone B (**53**) was previously identified in the soft coral *Dendronephthya* sp. [52].

In the South China Sea, research on the spiny-bodied sponge *Acanthella cavernosa* led to the discovery of three A-norsteroids: the ethyl esters of 2β-hydroxy-4,7-diketo-A-norcholest-5-en-2-oic acid (**61**), 24*S*-ethyl-2β-hydroxy-4,7-diketo-A-norcholest-5-en-2-oic acid (**62**), and 2β-hydroxy-4,7-diketo-24R-methyl-A-norcholest-5,22(*E*)-dien-2-oic acid (**63**) [53]. These steroids exhibited antifouling properties, showing effectiveness in inhibiting the settlement of *Balanus albicostatus*, with EC_50_ values of 8.2, 23.5, and 31.6 μg/mL, respectively.

Anthosterone B (**51**), along with two other A-norsteroids, crellasterones A (**64**) and B (**65**), was isolated from the sponge *Crella incrustans*, collected in New Caledonia [54]. Both crellasterones structurally resemble the semi-synthetic steroid maltadiolone (**66**), which is used medically and possesses calcium-binding activity comparable to cells not treated with calcium channel blockers [55]. This category also includes xidaosterol B (**67**), found in extracts from the South China Sea sponge *Neopetrosia chaliniformis* [56]. Furthermore, an extract from the brown alga *Sargassum carpophylum*, collected from the South China Sea, contained 24-ethyl-3-carbethoxy-3-hydroxy-A-norcholesta-5,24(28)-dien-2-one (**68**) [57]. A range of 3β-hydroxy-methyl-A-norsterane steroids (**69–75**) was isolated from the Okinawan marine sponge *Hymeniacidon aldis* [58]. The sponge *Hymeniacidon perlevis* (order Halichondrina) was found to contain significant amounts of stanols with a 3β-hydroxymethyl-A-nor-sterane nucleus (**18–25**), alongside sterols **28** and **70** [59].

A-norsteroids **28**, **72**, **76**, and 3β-(hydroxymethyl)-A-nor-5α-cholest-15-ene (**77**) were extracted from a *Clathria* sp. marine sponge, collected from southeast Sulawesi, Indonesia. Among these, clathruhoate (**76**), identified as 3β-(butyryloxymethyl)-A-nor-5α-cholestane, is noteworthy [60]. The Australian sponge *Phakellia aruensis* was the source of A-norsterols with various nuclei, specifically **19**, **21**, **78**, and **79** [61]. In a *Cribrochalina* sp. marine sponge, two A-norsteroids, **80** and haplosamate B (**81**), were discovered. Notably, haplosamate B exhibits inhibitory effects on membrane-type matrix metalloproteinase, with an IC_50_ value of 160 μg/mL [62].

Steroids **82**, **83**, and **84** were identified in extracts from marine sponges such as *Acanthella aurantiaca*, *Axinella tenuidigitata*, *Homaxinella trachys*, and *Phakellia aruensis* [39,40,41,42]. Additionally, 22,23-dihydro-A-norsteroids **85–90** were isolated from various marine sponges including *Acanthella aurantiaca*, *Acanthella cristagalli*, *Axinella verrucose*, *Homaxinella trachys*, *Hymeniacidon aldis*, *Phakellia aruensis*, *Pseudaxinyssa cantharella*, *Stylotella agminata*, and *Teichaxinella morchella* in diverse parts of the World Ocean [39,40,41,42,43,58,61]. The marine sponge *Acanthella cristagalli* contains 3-hydroxymethyl-24-ethyl-A-norcholest-7-ene (**91**) [63].

In the waters of Derawan Island, Indonesia, the marine sponge *Axinella carteri* was found to contain a moderately cytotoxic norsterol **92** [64]. The sponge *Haliclona oculata* yielded unusual A-norsteroids (**93–96**) [65]. From *Axinella proliferans*, collected in the Indian Ocean, several A-norsterols (**27**, **28**, **30**, **31**, **36**, **37**) were isolated, including rare nor-sterols with D-ring unsaturation, **77**, and 3β-(hydroxymethyl)-A-nor-5α-cholest-14-ene-16α-ol (**97**) [66]. A sea sponge from the Fiji Islands, *Spongia* sp., is the source of furanoditerpenoids named 3-nor-spongianones A (**98**) and B (**99**), which are unique due to their novel norspongian carbon skeleton, akin to A-ring-contracted nortriterpenoids [67].

A-nor-hippuristanol, A-nor-22-epi-hippurin-2α-carboxylic acid (**100**), was isolated from the gorgonian coral *Isis hippuris* [68]. Additionally, other soft corals from *Dendronephthya* sp. in the Nephtheidae family produced steroid **101** [69].

## 3. A-Norsteroids and Triterpenoids Derived from Terrestrial Sources

A-norsteroids and triterpenoids, derived from terrestrial sources, represent two distinct classes of naturally occurring organic compounds, each exhibiting unique structural characteristics and properties. Found in various terrestrial plants and fungi, A-norsteroids, though less common than other steroids, demonstrate significant biological activities and potential therapeutic applications. These applications include hormone therapy and anti-inflammatory treatments [1,2,3,4,5,11,12,70,71]. 

Triterpenoids, on the other hand, are prevalent in the plant kingdom and exhibit a broad distribution among terrestrial plants. As one of the largest and most diverse classes of plant metabolites, triterpenoids have been extensively researched for their pharmacological properties, including anti-inflammatory, antiviral, antibacterial, and anticancer effects. They are also utilized in traditional medicine and as natural supplements [3,4,11,12,70,71].

Both A-norsteroids and triterpenoids are crucial in the fields of natural product chemistry and pharmacology due to their varied structures and biological activities. They are key subjects in drug development and therapeutic applications. However, their efficacy and safety profiles require further research to fully comprehend their potential in medicine and other uses.

*Viburnum dilatatum*, commonly known as linden viburnum, is a deciduous shrub from the Adoxaceae family, introduced in the mid-Atlantic regions of the USA, from New York to Virginia. A notable feature of this shrub is its production of clusters of red drupes when ripe. Traditional Chinese medicine uses the berries, leaves, and stems to create remedies for snake bites, dysentery, and as an anthelminthic. From the leaves of this shrub, A-nortriterpenoids viburnols E (**102**), F (**103**), and G (**104**, structures in Figure 4) have been isolated [72,73].

The fruits of *Citrullus colocynthis* contain a cucurbitane 3-nor-triterpenoid named norcolocynthenin B (**105**), featuring a unique 5/6/6/5-fused-ring system. This compound exhibited significant cytotoxic activity against human cancer cell lines HL-60 (IC_50_ = 6.49 μM) and PC-3 (IC_50_ = 13.42 μM) [74].

*Dysoxylum hainanense*, a plant known for its twigs and leaves, is a source of triterpenoids like dysoxyhainic acids with a contracted five-membered A ring. Dysoxyhainic acid A (**106**), possessing an unprecedented 2-nor-1,3-cyclotirucallane skeleton, showed moderate antibacterial activity against Gram-positive bacteria [75].

The fruits of *Citrullus colocynthis* were also analyzed phytochemically, revealing the presence of structurally diverse nonanorcucurbitane-type triterpenoids, including colocynins, one of which is the bioactive colocynin A (**107**). This triterpenoid exhibited antiacetylcholinesterase activity and significant cytotoxicity against PACA, A431, and HepG2 cells [76,77].

Three triterpenoids, cucurbalsaminones A (**108**), B (**109**), and C (**110**), were isolated from a methanol extract of *Momordica balsamina*. These compounds feature a unique 5/6/3/6/5-fused pentacyclic carbon skeleton, named cucurbalsaminane, and displayed potent multidrug resistance (MDR)-reversing activity [78].

In a study of *Withania aristata*, two withanolide-type steroids were identified: 4β-formyl-6β,27-dihydroxy-1-oxo-witha-2,24-dienolide (**111**) and 4β-formyl-6β,27-dihydroxy-1-oxo-witha-24-enolide (**112**). These compounds demonstrated potent antiproliferative activity, inducing apoptosis in human tumor cells [79]. Additionally, an unusual withanolide glucoside with two cyclobutene rings, named trichoside B (**113**), was discovered in the *n*-butanol fraction of a 75% methanolic extract of the aerial parts of *Tricholepis eburnean* [80].

From the rhizomes of *Isodon amethystoides*, a triterpenoid named amethystoidesic acid (**114**) was isolated. This acid, with an unprecedented 5/6/6/6 tetracyclic skeleton, is the first triterpenoid of its kind, derived from a contracted A ring and the 18,19-seco-E-ring of ursolic acid, and exhibited inhibitory effects on nitric oxide production [81].

The triterpenoid gilvsin D (**115**), produced by the fruiting body of the pathogenic fungus *Phellinus gilvus*, known in traditional Chinese medicine as sanghuang, is used to alleviate abdominal pain and treat cancer [82]. Additionally, the unique A-nor-B-homosteroid **116**, containing a 10(5→4)-abeo-ergostane fragment, was isolated from the culture of basidiomycete *Polyporus ellisii* [83].

3-Hydorpxymethyl-A-nor cholestane (**117**) was found in extracts of Cretaceous black shales of Upper Barremian age (115 Myr BP) and is believed to be a component of sea sponges [84].

From *Urceola quintaretii*, two C19 steroids named urceoloids A (**118**) and B (**119**), featuring a unique spiro[4.4]nona-3,6,8-triene system, were isolated. These compounds exhibited immunosuppressive activities [85].

Lastly, three nortriterpenes from the roots of *Ziziphus mauritiana*, zizimauritic acids A (**120**), B (**121**), and C (**122**), with a unique A-nor-*E*-seco spiro-lactone ceanothane-type triterpene skeleton, were identified. These compounds displayed cytotoxicity, with IC_50_ values ranging from 5.05 to 11.94 μg/mL, and compounds **120** and **122** inhibited the growth of *Staphylococcus aureus*, with IC_50_ values of 2.17 and 12.79 μg/mL, respectively [86].

In the study of *Dysoxylum hainanense*, a novel triterpenoid named dysoxyhainol (**123**, structures in Figure 6) was identified in its twigs and leaves. This compound, with a modified A-ring structure, exhibited moderate antibacterial activity against Gram-positive bacteria [75]. Additionally, the extraction of *Ceanothus americanus*, commonly known as Jersey tea, yielded a pentacyclic C29 triterpenoid named A-norlupa-1,22-diene-14,17-dicarboxylic acid or ceanothenic acid (**124**), along with ceanothic acid (**126**) [87]. Another compound, ceanothanic acid (**125**), was isolated from the roots of *Ziziphus mauritiana* [86,87]. 

From the roots of *Ziziphus jujuba*, a cytotoxic compound, 2,28-Dinor-24-hydroxylup-1,17(22)-dien-27-oic acid (**127**), was discovered [88,89,90]. Jingullic acid (**128**), another noteworthy compound, was found in the bark of *Emmenosperma alphitoniodes* [91].

Granulosic acid (**129**) was detected in ether extracts of the heartwood of Colubrina granulosa [87]. Zizyberenalic acid (**130**) has been identified in various parts of the *Ziziphus* genus, including *Z. jujuba* fruit, *Z. cambodiana* roots and bark, and *Z. jujuba* roots [88,90,92]. From the roots of *Z. jujube*, two unique compounds, musancrpic acids A (**131**) and B (**132**), featuring an *E*-ring clactone structure, were isolated [90].

The roots of *Coleus forskohlii* yielded a rearranged pentacyclic triterpenoid, 2-hydroxy-methyl A-(1)nor-urs-19α-hydroxy-2(3),12(13)dien-28-oic acid or coleonolic acid (**133**) [93]. Additionally, the whole plant of *Agrimonia pilosa* led to the discovery of a nortriterpenoid named 19α-hydroxy-2-oxo-nor-A(3)-urs-11,12-dien-28-oic acid, referred to as agrimo-norterpene A (**134**) [94].

Melantheraside E (**135**), a 3-oxo-2β-carboxyamino-12α-chloro,13β-hydroxy-1-nor-oleanan-28,13-olide, was found in the aerial parts of *Melanthera elliptica* [95]. From *Davidia involucrata*, the A-ring-contracted nortaraxerane davinolunone A (**136**) was isolated [96]. This plant also yielded two taraxerene-type triterpenes, 2-nor-D-friedoolean-14-en-28-ol (**137**) and 2-nor-D-friedoolean-14-en-3α,28-diol (**138**), which demonstrated moderate cytotoxicity against various cancer cell lines [97].

The roots of *Rubus innominatus* produced two nortriterpenes, rubuminatuses A (**139**) and B (**140**), each containing a unique contracted five-membered A-ring ursane-type skeleton. These compounds exhibited significant inhibitory effects on cytokines [98]. From *Salvia buchananii* roots, a pentacyclic triterpene called hyptadienic acid (**141**) was isolated, inducing an S cell cycle block in HeLa cells [99]. In the roots of *Potentilla freyniana*, A-ring-contracted triterpenoids were detected, including madengaisu B (**142**), rosamultic acid (**143**), hyptadienic acid (**144**), madengaisu A (**145**), and sculponeatic acid (**146**) in 95% EtOH extracts [100]. Madengaisu B and hyptadienic acid showed cytotoxic activity against BGC-823 and HepG2 cells, respectively.

**Figure 7:**
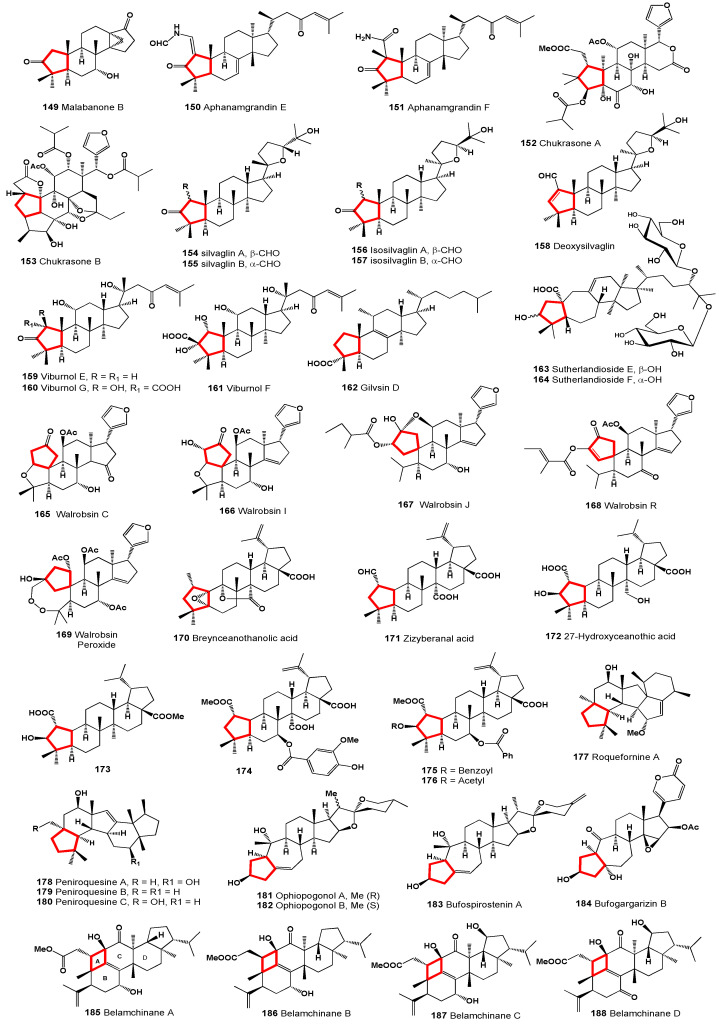
A-norsteroids isolated from extracts of plants or their individual parts, roots, bark, leaves.

*Dysoxylum hainanense* twigs and leaves metabolized A-ring-modified triterpenoids, namely dysoxyhainic acids B (**147**) and C (**148**), alongside dysoxyhainol (**123**), all showing moderate antibacterial activity against Gram-positive bacteria [75], and from *Ailanthus malabarica* stem bark, a triterpenoid named malabanone B (**149**, structures in Figure 7), featuring a unique tricyclo[4.3.1.01,6]decane unit, was isolated [101]. *Aphanamixis grandifolia* stems yielded aphanamgrandins E (**150**) and F (**151**), which are 2,3-seco-tirucallane triterpenoid derivatives [102]. *Chukrasia tabularis* produced two limonoids, chukrasones A (**152**) and B (**153**), exhibiting potential inhibition of the delayed rectifier (IK) K+ current [103].

*Aglaia sylvestris* rootwood provided dammarane-type triterpenoids silvaglin A (**154**), isosilvaglin A (**156**), their 1H-β-epimers silvaglin B (**155**) and isosilvaglin B (**157**), and deoxysilvaglin (**158**) with a Δ1(3)-bond [104]. *Viburnum dilatatum* leaves yielded dammarane triterpenoids viburnols E (**159**) [105,106], F (**161**), and G (**160**). The lanostane-type triterpenoid gilvsin D (**162**) was isolated from the mushroom *Phellinus gilvus* [107].

From the southern African legume *Sutherlandia frutescens*, two rearranged cycloartanol glycosides, sutherlandiosides E (**163**) and F (**164**), were isolated. These compounds are notable for their aglycones, which feature a unique rearranged five- and seven-membered A/B-ring system [108]. Additionally, several neotecleanin-type limonoids (**165–169**) were detected in methanol (MeOH) extracts of the root barks of *Walsura robusta* [109].

A nor-ceanothane-type triterpenoid, breynceanothanolic acid (**170**), was obtained from the roots of *Breynia fruticose*. This compound demonstrated moderate cytotoxicity against five human cancer cell lines [110,111]. In the fruits of *Ziziphus jujuba* (Rhamnaceae), 2α-aldehydo-A(1)-norlup-20(29)-en-27,28-dioic acid (zizyberanal acid, **171**) was identified [112].

27-Hydroxyceanothic acid (**172**) was found in the roots and bark of *Ceanothus americanus* [113], as well as in the roots of *Paliurus ramosissimus* [114]. The C-28 methyl ester of ceanothic acid (**173**) was detected in the bark of *Zizyphus joazeiro* [115] and in the seeds of *Zizyphus jujube* [116].

A jujubogenin glycoside, namely 3-O-acetylcolubrin (**174**), was isolated from the leaves of *Colubrina asiatica* [117]. Ether extracts from the heartwood of *Colubrina granulosa* yielded the polyphenolic coumaranone maesopsin 3,7-O,O-dibenzoyl ceanothic acid methylester (**175**) and 3-O-acetyl-7-O-benzoyl ceanothic acid methylester (**176**) [118].

Roquefornine A (**177**), a sesterterpenoid featuring an unprecedented 5/6/5/5/6-membered pentacyclic system, was characterized from the fungus *Penicillium roqueforti* YJ-14 [119]. Additionally, other sesterterpenes, peniroquesines A–C (**178–180**), with a unique 5–6–5–6–5-fused pentacyclic ring system, were isolated from the same fungus through solid fermentation [120,121].

The medicinal herb *Ophiopogon japonicus*, according to a 70% ethanol extract of its rhizomes, contained steroidal glycosides known as ophiopogonols. Notably, ophiopogonols A (**181**) and B (**182**) are rare spirostanols with a rearranged A/B-ring system (5/7/6/5/5/6-ring system) not previously identified in plants [122]. Bufospirostenin A (**183**), a steroid with rearranged A/B rings, was isolated from the toad *Bufo bufo gargarizans*. This compound is notable as the first spirostanol identified in animals [123]. A similar steroid, bufogargarizin B (**184**) with an unprecedented skeleton, was discovered in the venom of *Bufo bufo gargarizans* [124].

Belamchinanes A–D (**185–188**), four triterpenoids with a novel skeleton, were isolated from the seeds of *Belamcanda chinensis*. These structures feature a 4/6/6/6/5 polycyclic system, where a four-membered carbocyclic ring bridges the C-1 and C-11 positions of a classical triterpenoid framework [125].

Summarized information on biological activity of A-norsteroids is presented in Table 1, and details are described partially in the text or a full description of the activity is written in the original articles.

## 4. B-Norsteroids Derived from Marine Sources

B-norsteroids and abeo-steroids are two distinct types of modified steroids, each characterized by unique structural features. B-norsteroids involve a modification in the B ring of the steroid nucleus, where one carbon atom is absent compared to the standard steroid structure. This alteration can significantly impact the biological activity of the steroid, leading to unique pharmacological properties and potential applications in medicine, including contraceptive development and other therapeutic agents [3,5,12,18,35,36,37,41,42].

**Figure 8:**
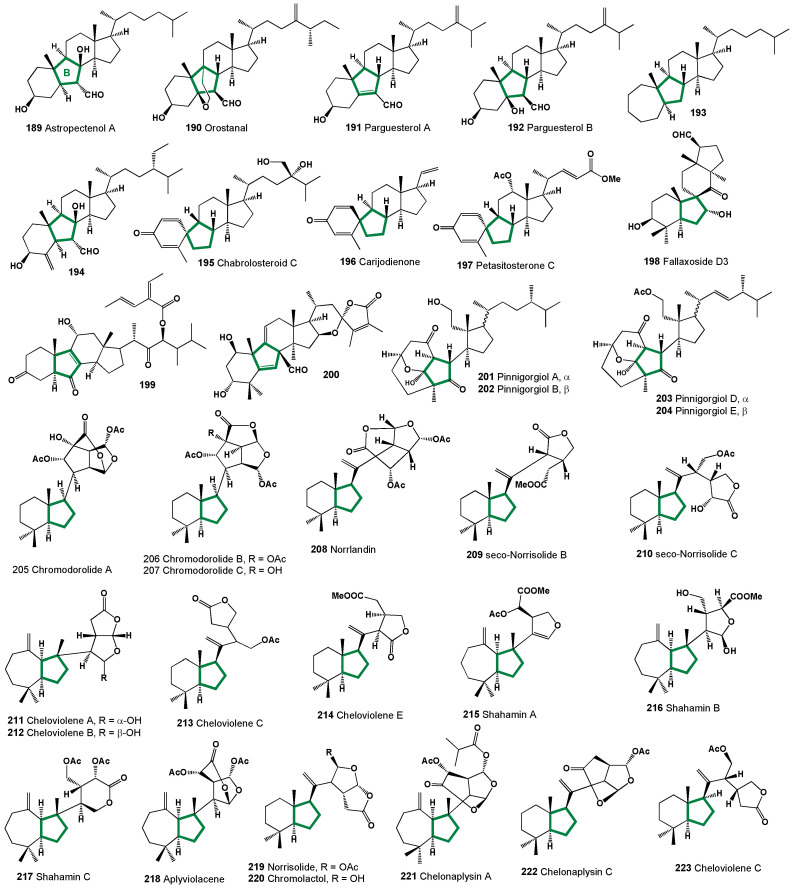
B-norsteroids isolated from marine invertebrate extracts.

**Figure 9:**
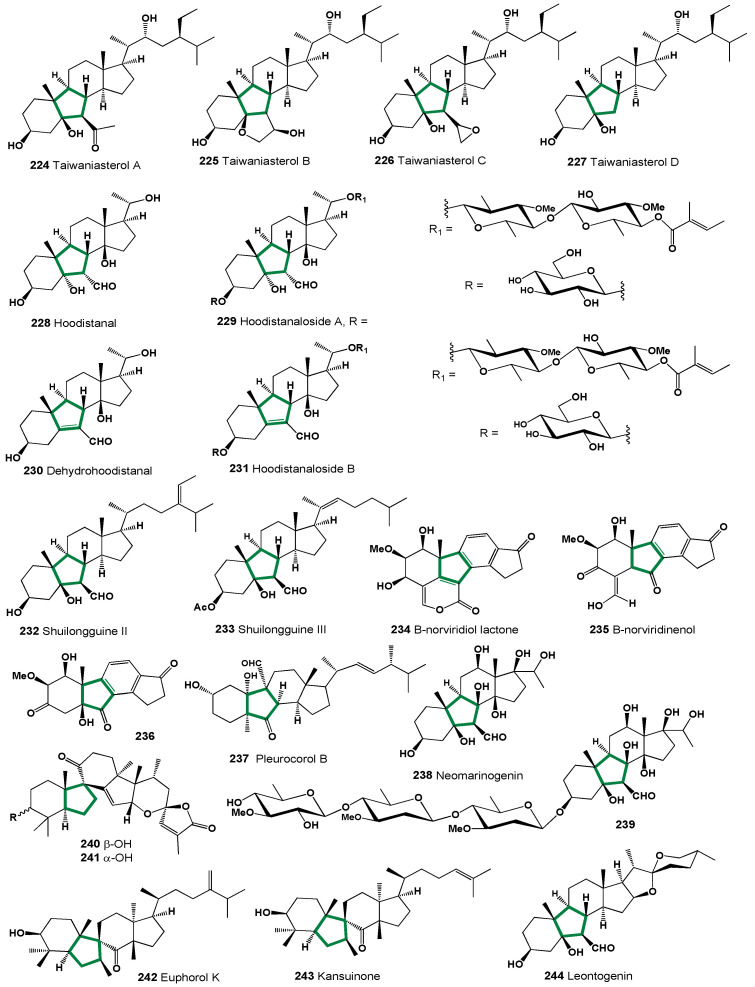
B-norsteroids isolated from plant extracts or their individual parts.

**Figure 10:**
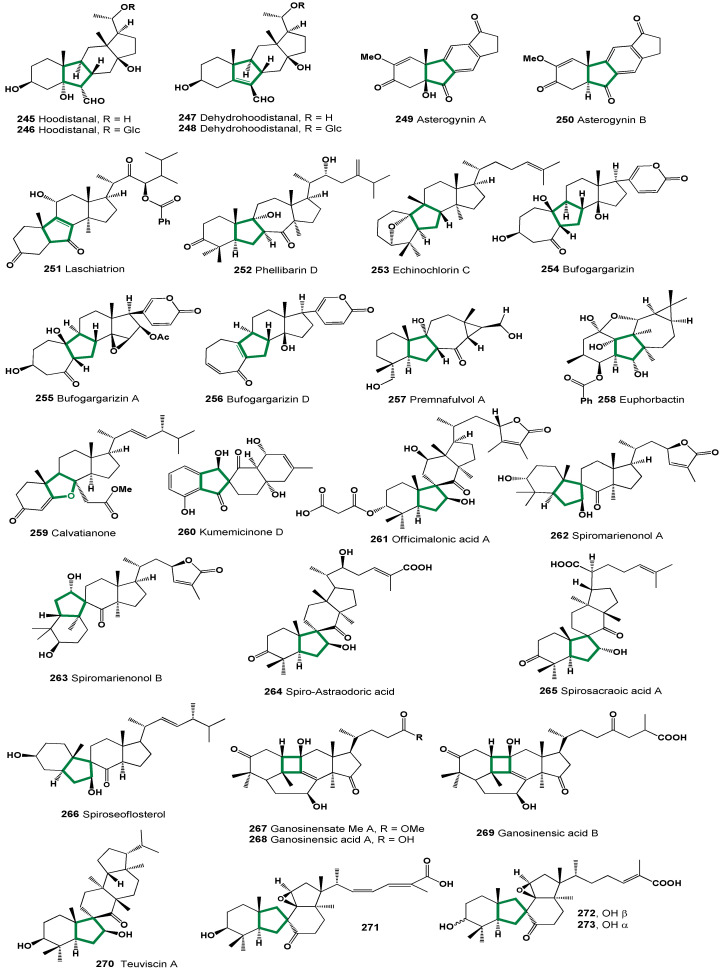
B-norsteroids isolated from extracts of plants, fungi, and amphibians.

**Figure 11:**
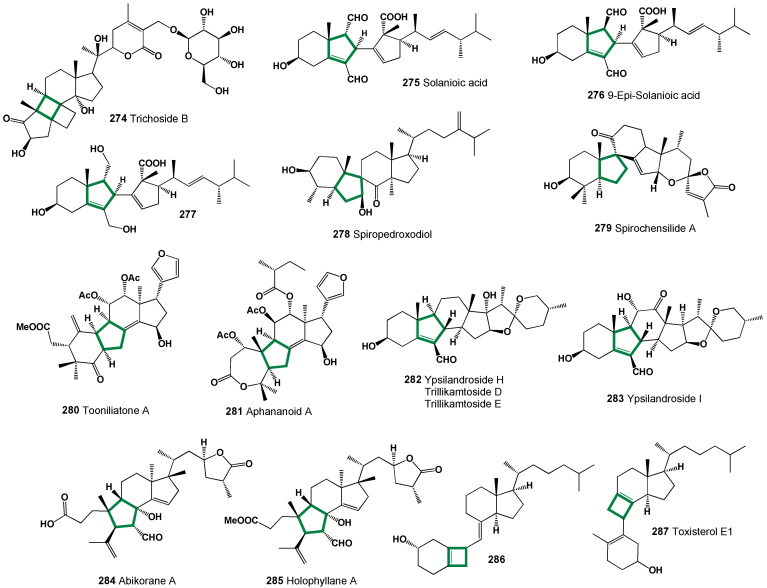
B-norsteroids and their derivatives isolated from extracts of plants and fungi.

Abeo-steroids, on the other hand, undergo a more drastic structural rearrangement than B-norsteroids. The term “abeo” signifies a deviation from the normal steroid structure, often involving the breaking and reforming of rings in the steroid nucleus. These rearrangements can result in substantial changes in the biological activity of the steroids, offering potential for unique therapeutic benefits. Like B-norsteroids, abeo-steroids have potential medicinal and pharmacological applications, though their development and study may be more complex due to significant structural changes [3,5,12,18,35,36,37,41,42]. The deformed B ring in the B-norsteroids (**189–287**) is highlighted in green in Figure 8, Figure 9, Figure 10 and Figure 11.

Both B-norsteroids and abeo-steroids are based on the core steroid structure, sharing the basic four-ring configuration common to all steroids. They are of significant interest in pharmacology and medicinal chemistry for their potential to yield new therapeutic agents, representing key structural modifications in steroid chemistry.

In the field of marine natural products, both B-norsteroids and marine-derived triterpenoids play a significant role. This research branch focuses on the ocean’s biodiversity for novel compounds that could lead to new or improved pharmaceuticals. Marine ecosystems’ unique conditions often produce structurally distinctive and biologically potent compounds not found in terrestrial environments [41,42,126,127,128,129].

From the methanolic extract of the starfish *Astropecten polyacanthus*, four steroids named astropectenols A–D were identified, but only astropectenol A (**189**, structures in Figure 7) possesses a B-nor skeleton. The CH_2_Cl_2_ fraction of this extract exhibited strong cytotoxic effects against human leukemia HL-60 cells [130,131]. A sterol derivative, 5(6→7)-abeo-sterol, orostanal (**190**), was obtained from an extract of the marine sponge *Stelletta hiwasaensis* and induced apoptosis in human acute promyelotic leukemia cells [132,133]. 

An antitubercular extract from the Caribbean Sea sponge *Svenzea zeai* yielded 5(6→7)-abeo-sterols, parguesterol A (**191**) and parguesterol B (**192**) [134]. A-homo-B-norsterol (**193**) has been identified among the volatile compounds in marine sediments [135,136,137]. 8β-Hydroxy-7α-formyl-B-northeonellasterol (**194**) was detected in MeOH and CH_2_Cl_2_ extracts from the marine sponge *Theonella swinhoei*, collected from the province of Bohol in the Philippines [138].

An unusual metabolite with a spiro-ring A, B system, known as spirosteroid chabrolosteroid C (**195**), was detected in the organic extract of the Taiwanese soft coral *Nephthea chabrolii* [139]. Carijodienone (**196**), featuring a spiro[4,5]decane core derived from an A–B-ring rearrangement of a steroidal nucleus, was isolated from the Pacific octocoral *Carijoa multiflora* [140]. Another spirosteroid, petasitosterone C (**197**), possessing a rare A/B spiro[4,5]decane ring system, was identified in the Formosan marine soft coral *Umbellulifera petasites* [141].

An unprecedented triterpene glycoside with the aglycone fallaxoside D3 (**198**) was found in the sea cucumber *Cucumaria fallax*, belonging to the Cucumariidae family in the Dendrochirotida order [142]. B-nortriterpenoids **199** and **200** were discovered in the extract of a cultivated fungus isolated from a marine sponge off the coast of Australia [143].

In recent years, increased attention has been given to secondary metabolites that undergo degradation, including B-norsteroids and B-nortriterpenoids. These compounds are associated with aging or other yet-to-be-understood biological processes. Several measurements of degradable steroids or triterpenoids have been identified in marine invertebrates. For instance, pinnigorgiols A–E (**201–204**), which are 9,11-secosteroids with a unique tricyclic γ-diketone framework, were obtained from the gorgonian coral *Pinnigorgia* sp. [144,145]. Highly degraded steroids, chromodorolide A (**205**), B (**206**), and D (**207**), were detected in a methanol (MeOH) extract from spongivorous nudibranchs of the genus *Chromodoris* and the marine sponge *Chromodoris* sp. collected along the coast of Okinawa [146]. The same chromodorolides A–C were isolated from the Australian sponge *Aplysilla sulphurea*. All chromodorolides exhibited significant cytotoxicity against the P388 mouse leukemia cell line and showed activity against the free-living larval stages of the parasitic nematodes *Haemonchus contortus* and *Trichostrongylus colubriformis* [147].

In the study of a Red Sea *Dysidea* species, four rearranged spongiane-type diterpenes were isolated: norrlandin (**208**), seco-norrisolide B (**209**), seco-norrisolide C (**210**), and norrisolide (**219**). Both norrlandin and norrisolide exhibited cytotoxic activities [148]. Norrisolide (**219**), in particular, has been identified as a phospholipase A inhibitor, possessing anti-inflammatory and ichthyotoxic properties. It was found in various marine organisms, including *Chromodoris norrisi*, *Chelonaplysilla violacea*, *Dendrilla* sp., and *Dysidea* spp. [149,150,151,152]. The New Zealand sponge *Chelonaplysilla violacea* contains rearranged sponginess, including cheloviolenes A (**211**), B (**212**), C (**213**), E (**214**), and norrisolide (**216**) [152].

An investigation into two Red Sea *Dysidea* sponges yielded rearranged spongian degraded steroids shahamins A (**215**), B (**216**), and C (**217**), along with aplyviolacene (**218**) [148,153]. Aplyviolacene (**218**) was also detected in the Australian sponge *Chelonaplysilla violacea* [154]. Two rearranged spongiane-type diterpenes, chelonaplysins A (**221**) and C (**222**), identified from the Pohnpeian marine sponge *Chelonaplysilla* sp., demonstrated antimicrobial activity against *Bacillus subtilis* [155]. Additionally, chromolactol (**220**) and cheloviolene C (**223**) were found in extracts of the Indo-Pacific nudibranch *Goniobranchus coi* [156].

## 5. B-Norsteroids and Triterpenoids Derived from Terrestrial Sources

B-norsteroids, like many steroids, are derived from both natural and synthetic sources. Naturally, they are found in various plants and animals, often playing roles in the organisms’ biology, typically as part of defense mechanisms. In the laboratory, chemists can modify naturally sourced steroids or synthesize B-norsteroids from basic organic chemicals using complex chemical processes [12,18,35,36,37,41,42,157,158].

Taiwaniasterols A (**224**), B (**225**), C (**226**), and D (**227**), with a unique 6–5–6–5-fused-ring skeleton, were isolated from the leaves of *Taiwania cryptomerioides* [159]. *Hoodia gordonii*, a succulent from the Kalahari Desert, has been used traditionally for various ailments and as an appetite suppressant. The analysis of its aerial parts led to the isolation of active pregnane glycosides, including hoodistanal (**228**, structures in Figure 8), dehydrohoodistanal (**230**), and hoodistanalosides (**229** and **231**) [160].

An ethanolic extract of *Polypodium niponicum* (Chinese name “shuilonggu”) contained two steroids, shuilongguine II (**232**) and shuilongguine III (**233**) [161]. Viridin-related B-norsteroids, B-norviridiol lactone (**234**) and B-norviridin enol (**235**), both with unique unprecedented carbon skeletons, were isolated from *Hymenoscyphus pseudoalbidus*, a fungus responsible for ash dieback. Compound **235** degraded to another B-norsteroidal compound, 1β-hydroxy-2α-hydro-asterogynin A (**236**), later detected in the original culture [162].

The fruiting bodies of *Pleurotus cornucopiae* contain pleurocorol B (**237**) with an unprecedented carbon skeleton [163]. Neomarinogenin (**238**) and its C21 steroidal glycoside (**239**) were isolated from *Marsdenia incisa* [164]. Spirochensilides A (**240**) and B (**241**), unique triterpenoids with an 8,10-cyclo-9,10-seco and methyl-rearranged skeleton, were isolated from *Abies chensiensis* [165].

Euphorol K (**242**) was found in the water–methanol extract of the dried latex of *Euphorbia resinifera*, exhibiting cytotoxic effects against MCF-7 [166]. Kansuinone (**243**), a rearranged euphane triterpenoid with a spiro[5,6]-ring system from *Euphorbia kansui* roots, showed inhibitory activity against 11β-HSD1 [167]. A B-ring-contracted spirostane, leontogenin, 25(R)-B-nor(7)-6β-formyl-spirostane-3β,5β-diol (**244**), was isolated from an acid hydrolysate extract of *Tacca leontopetaloides* leaves [168].

*Hoodia gordonii*, a succulent native to the Kalahari Desert, is known for its appetite-suppressing properties and has gained popularity in Western countries as a dietary supplement. This plant contains B-norsteroids, including hoodistanal (**245**), dehydrohoodistanal (**247**), and their glycosides, hoodistanalosides A (**246**) and B (**248**) [160].

An endophytic fungus from the small palm *Asterogyne martiana* produced two unusual steroid-like metabolites, asterogynin A (**249**) and asterogynin B (**250**), with potential antimalarial properties [169]. The antibiotic laschiatrion (**251**), isolated from the orange pore fungus *Favolaschia* sp. 87129, exhibited broad in vitro activity against several human pathogens [170].

Phellibarin D (**252**), a B/C-ring-rearranged lanostane triterpenoid with a 6/5/7/5-ring framework, was isolated from *Phellinus rhabarbarinus*. This compound showed cytotoxicity against human cancer cell lines and inhibited nitric oxide production in macrophages [171].

Echinochlorin C (**253**) detected in methanol extracts of *Echinochloa utilis*, displayed potential cytotoxic activity against HeLa cells, indicating possible therapeutic applications for cancer treatments [172].

Bufogargarizin (**254**) was isolated from chan su, a traditional Chinese medicine used in cancer treatment, showing weak cytotoxic activities [173]. Bufogargarizin A (**255**) with an unprecedented skeleton was discovered in the venom of *Bufo bufo gargarizans* [124], and bufogargarizin D (**256**) was found in the eggs of the same toad [174].

*Premna fulva*, used in Zhuang medicine, contained a unique metabolite, premnafulvol A (**257**), with a 6/5/7/3-fused tetracyclic carbon skeleton, isolated from its aerial parts [175]. Euphorbactin (**258**), a terpenoid with a similar fused-ring skeleton, was isolated from *Euphorbia micractina* [176].

Calvatianone (**259**), a sterol with a 6/5/6/5-fused ring system and a contracted tetrahydrofuran B ring, was detected in *Calvatia nipponica* [177]. Kumemicinone D (**260**), a B/C-ring-rearranged product, was discovered in the deep-sea actinomycete *Actinomadura* sp. KD439, showing cytotoxicity against leukemia cells [178].

Officimalonic acid A (**261**), a 24-methyl-lanostane triterpene with a unique skeleton, was isolated from *Fomes officinalis* [179]. Spiromarienonols A (**262**) and B (**263**), triterpene lactones with unique structures, were isolated from *Abies mariesii* Masters [180].

Spiro-astraodoric acid (**264**), a lanostane-type triterpenoid with a spirocyclic structure, was discovered in *Astraeus odoratus*, exhibiting cytotoxicity against various cancer cell lines [181]. Spirosacraoic acid A (**265**), with a rearranged tirucallane skeleton, was isolated from *Boswellia sacra* [182].

Spiroseoflosterol (**266**), a unique ergostane steroid, was isolated from *Butyriboletus roseoflavus*, showing cytotoxicity against liver cancer cell lines [183]. Methyl ganosinensate A (**267**), ganosinensic acid A (**268**), and ganosinensic acid B (**269**), triterpenoids with a four-membered ring, were isolated from *Ganoderma sinense* [184].

Teuviscin A (**270**), a triterpenoid with a rare 7(8→9)-abeo-9R-D:C-friedo-B’:A’-neo-gammacerane skeleton, was isolated from the whole plants of *Teucrium viscidum* [185]. Additionally, other triterpenes (**271**, **272**, and **273**, structures in Figure 11) featuring an 11(10→8)-abeo-lanostane carbon skeleton were extracted from the bark of *Garcinia speciosa* [186].

The aerial parts of *Tricholepis eburnea*, collected from the Ziarat Valley in Pakistan’s province of Balochistan, contained a unique withanolide glucoside named trichoside B (**274**, structures in Figure 11). This compound was found in the *n*-butanolic fraction of a 75% methanolic extract [187], and it has also been previously identified in the plant *Gypsophila trichotoma* [188].

Solanioic acid (**275**), a degraded and rearranged steroid with in vitro antibacterial activity against methicillin-resistant *Staphylococcus aureus* (MRSA), was isolated from laboratory cultures of the fungus *Rhizoctonia solani*, sourced from tubers of *Cyperus rotundus* collected in Sri Lanka [189]. Subsequently, 9-epi-solanioic acid (**276**) and another compound (**277**) were discovered in the same fungus [190], and from the flowering plant *Euphorbia pedroi*, a tetracyclic triterpenoid with an unusual spiro scaffold, spiropedroxodiol (**278**), was isolated. This compound proved to be a potent MDR reversal agent in L5178Y-MDR and Colo320 cells [191]. A triterpenoid, spirochensilide A (**279**), with a unique 8,10-cyclo-9,10-seco and methyl-rearranged carbon skeleton, was found in extracts from the leaves of *Abies chensiensis* [192]. Tooniliatone A (**280**), a limonoid with an unprecedented 6/5/6/5 tetracarbocyclic skeleton, was isolated from *Toona ciliata* var. *yunnanensis* [193]. Another limonoid, aphananoid A (**281**), featuring a rare C24 appendage and a 5/6/5-fused-ring framework, was obtained from *Aphanamixis polystachya*. This compound inhibited nitric oxide production in the RAW2647 cell line [194].

N-norsteroids, featuring novel 5(6 → 7)-abeo-steroidal aglycones, ypsilandrosides H (**282**) and I (**283**), were obtained from the whole plants of *Ypsilandra thibetica* [195]. Additionally, two glycosides, trillikamtoside D and trillikamtoside E with aglycone structures similar to ypsilandroside H (**282**), were detected in the hemostatic fraction of the 75% aqueous ethanol extract of *Trillium kamtschaticum* [196].

Abikorane A (**284**), a nor-3,4-seco-17,14-friedo-lanostane triterpenoid isolated from the leaves of *Abies koreana*, showed strong cytotoxic activities against several cancer cell lines (A549, SK-OV-3, SK-MEL-2, and HCT-116) with IC_50_ values ranging from 0.89–9.62 μM. It also inhibited lipopolysaccharide-stimulated nitric oxide production (IC_50_ value of 11 μM) and exhibited a significant nerve growth factor release effect (192%) from C6 glioma cells [197]. Holophyllane A (**285**), a triterpenoid with a B-nor-3,4-seco-17,14-friedo-lanostane structure, was isolated from the methanol extract of *Abies holophylla* trunks. It displayed moderate to weak cytotoxicity and significant inhibitory activity against nitric oxide (NO) production [198].

Two photoproducts of vitamin D were identified during photochemical processes. One, a cyclobutane-containing derivative (**286**), was identified [199], and a similar secosteroid, toxisterol E1 (**287**), a minor transformation product of vitamin D2, was found in various mushrooms [200]. Both steroids exhibit antihypercholesterolemic and hypolipidemic activities and act as inhibitors of cholesterol synthesis [1].

A summary on the biological activity of B-norsteroids is presented in Table 2, and details are described partially in the text or a full description of the activity is written in the original articles.

## 6. C-Norsteroids Derived from Marine and Freshwater Sources

C-norsteroids, characterized by a missing carbon atom(s) in the C ring of the steroid nucleus, are fascinating compounds produced by marine organisms like sponges, corals, and others. These unique molecules, often with significant biological activities, are products of the diverse marine environment, rich in ecological niches and biological interactions [4,5,7,12,18,21].

C-norsteroids from marine sources display distinct biological properties due to their altered structures, which may include specific interactions with biological receptors, enzymes, or other targets. These properties are of keen interest in pharmacology and natural product chemistry, as they offer potential for new therapeutic applications, particularly in areas where conventional treatments are less effective [5,7,12,18,21,201,202,203]. 

An interesting fact is that in many C-norsteroids, as the C ring decreases, the D ring increases from five-membered to six-membered or seven-membered. The fact is indeed fascinating, especially in the context of organic chemistry and biochemistry [16,17,199,201]. In chemical structures, ring tension plays a significant role in the stability of the molecule. Typically, five- and six-membered rings are more stable and less strained than smaller or larger rings due to the angles between the atoms being closer to ideal tetrahedral angles (109.5°). So, a change in ring size can significantly impact the molecule’s stability and reactivity. The change in ring size can alter the steric (spatial) and electronic (distribution of electrons) properties of the molecule [16,17,199,201,203]. 

**Figure 12:**
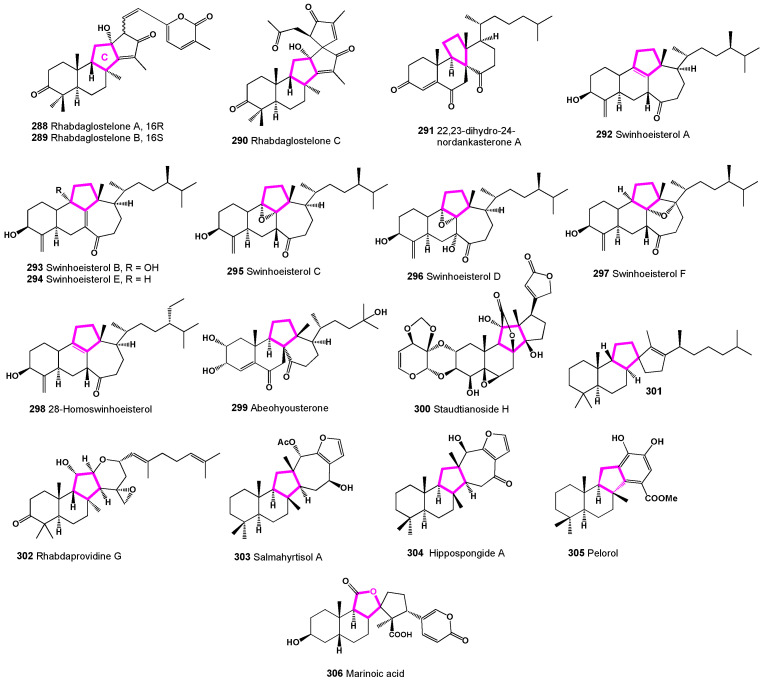
C-norsteroids and triterpenoids derived from marine and freshwater organisms.

**Figure 13:**
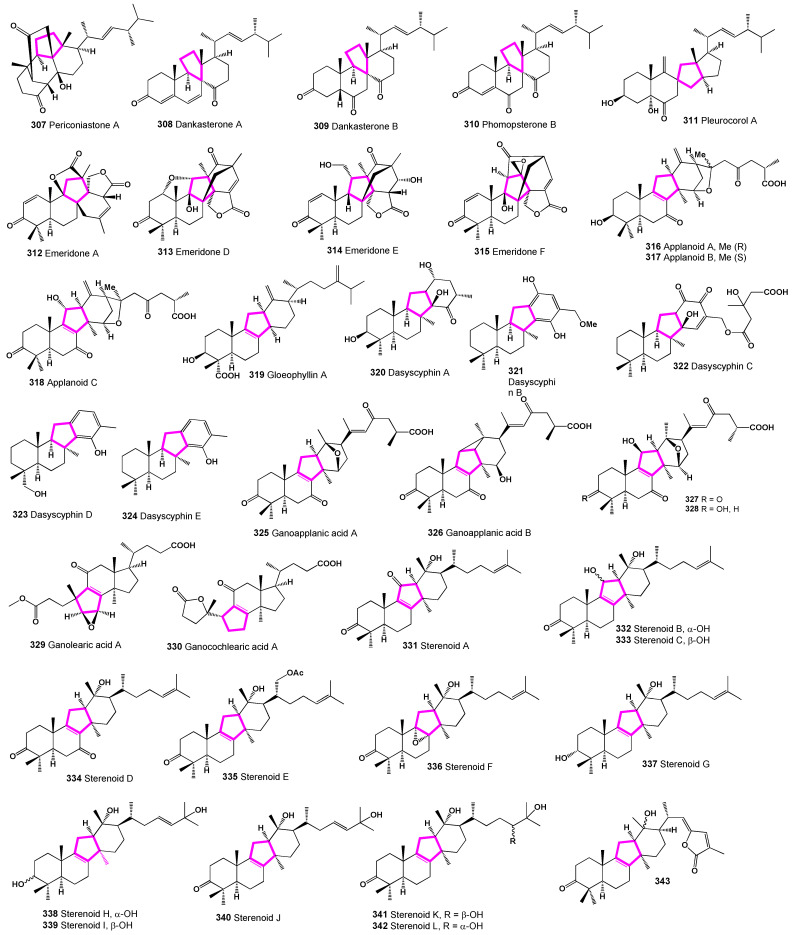
C-norsteroids produced by fungi and isolated from their extracts.

**Figure 14:**
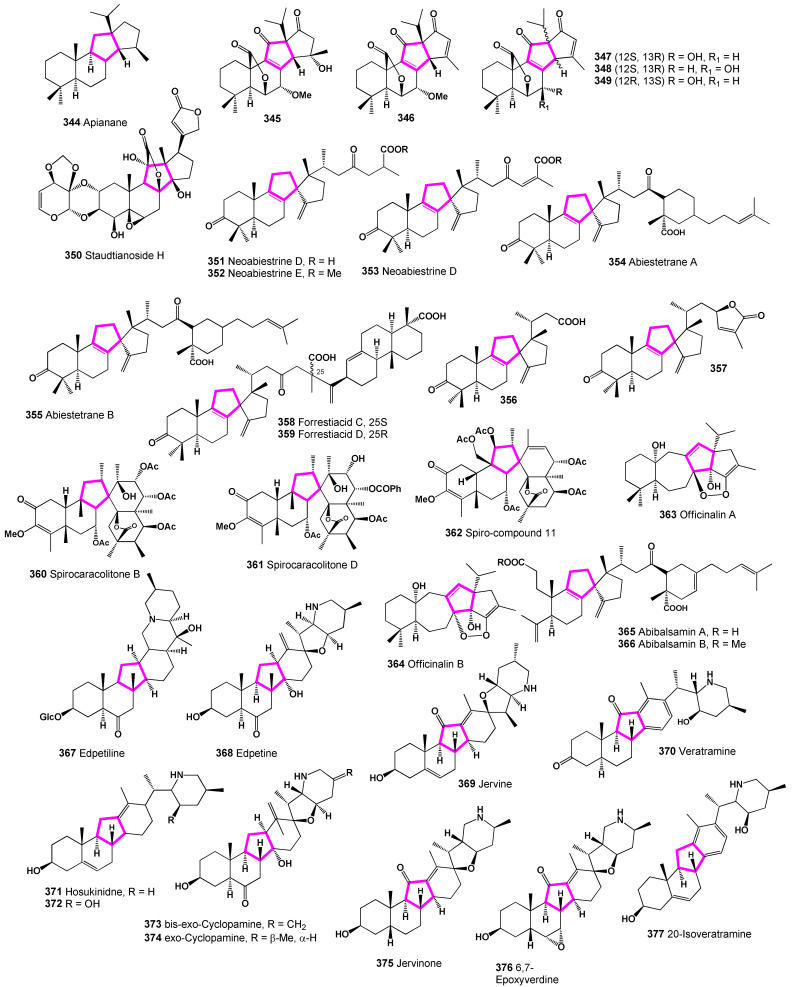
C-norsteroids isolated from plant extracts.

**Figure 15:**
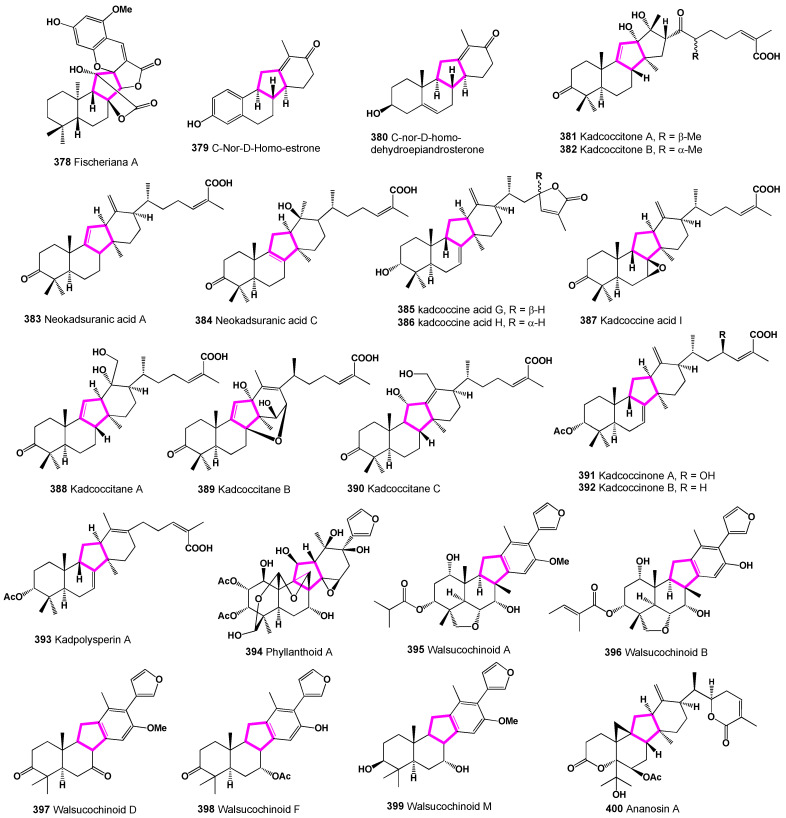
C-norsteroids isolated from plant extracts.

**Figure 16:**
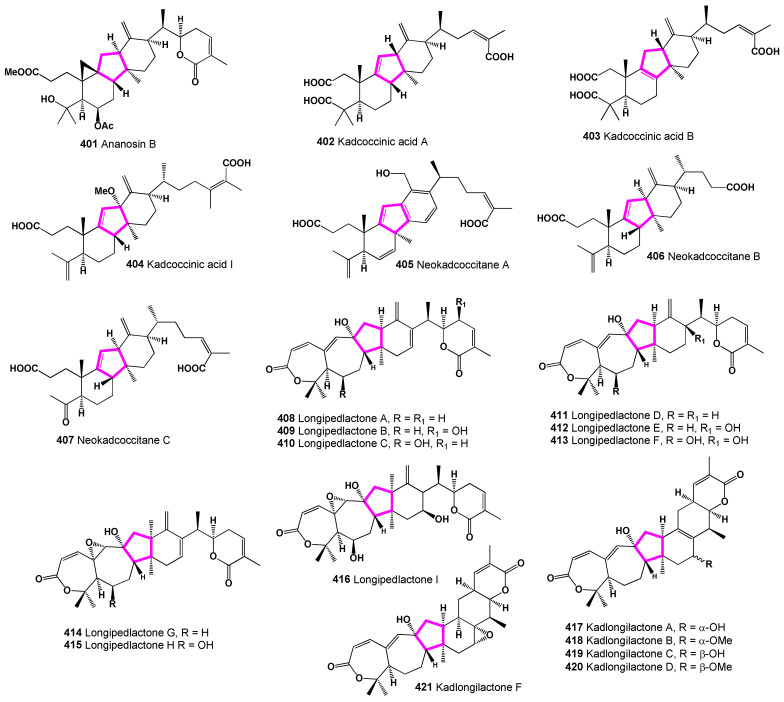
C-norsteroids derived from plants or individual parts of leaves, fruits.

This can affect how the steroid interacts with other molecules, such as receptors or enzymes, influencing its biological activity. In a biological context, the alteration in ring size might reflect an evolutionary adaptation. Different ring sizes could lead to different biological functions or interactions within organisms. Overall, the interplay between ring tension, molecular stability, and biological function in the context of changes in ring size in C-norsteroids is a remarkable example of the complexity and elegance of organic molecules. The deformed C ring in the C-norsteroids (**288–421**) is highlighted in pink in Figure 12, Figure 13, Figure 14, Figure 15 and Figure 16. 

The exploration of C-norsteroids is an integral part of the search for novel compounds in drug discovery. Their unique structural features, especially the modifications to the steroid nucleus, are of particular interest for their potential novel mechanisms of action or improved pharmacological profiles. For example, the tetracyclic and pentacyclic isomalabaricanes named rhabdaglostelones A (**288**), B (**289**), and C (**290**) from the marine sponge *Rhabdastrella globostellata* showed cytotoxic activities toward various cancer cell lines [204]. 22,23-dihydro-24-nordankasterone A (**291**) from the sponge *Luffariella variabilis* exhibited cytotoxicity towards breast cancer cells [205].

The sea sponge *Theonella swinhoei* produces swinhoeisterols A–F (**292–297**, and **298**) with an unprecedented 6/6/5/7-ring system. These compounds have shown cytotoxicity towards lung and bone cancer cells, with compound **294** exhibiting an inhibitory effect on (h)p300 [206,207]. Abeohyousterone (**299**, structures in Figure 12) isolated from the Antarctic tunicate *Synoicum adareanum* demonstrated moderate cytotoxicity against several cancer cell lines [208].

Akadisulfate A (**300**), a sulfated meroterpenoid with a hydroquinone moiety from the sponge *Aka coralliphaga*, and C-norsteroid **301**, found in crude oil, represent the diversity of these compounds [209,210,211]. Rhabdaprovidine G (**302**), from the Vietnamese sponge *Rhabdastrella providentiae*, and salmahyrtisol A (**303**), from the Red Sea sponge *Hyrtios erecta*, showed significant cytotoxicity against various cancer cell lines [212,213,214]. Hippospongide A (**304**), similar to salmahyrtisol A, was isolated from *Hippospongia* sp. [215].

Pelorol (**305**), an aromatic substituted sesquiterpene from the tropical marine sponge *Dactylospongia elegans*, exhibited antitrypanosomal and antiplasmodial effects [216]. Marinoic acid (**306**), found in the skin of the toad *Bufo marinus*, showed inhibition of Na+ and K+-ATPase enzymatic activity [217]. These findings underscore the rich potential of marine-derived C-norsteroids and related compounds in developing new pharmacological agents.

## 7. C-Norsteroids Derived from Fungi and Fungal Endophytes

Periconiastone A (**307**), an ergosterol with an unprecedented pentacyclo-[8.7.0.01,5.02,14.010,15]-heptadecane system, was isolated from the fungus *Periconia* sp. TJ403-rc01. It exhibited antibacterial activity against Gram-positive *S. aureus* and *E. faecalis* with MIC values of 4 and 32 μg/mL, respectively [218]. Dankasterone A (**308**) and dankasterone B (**309**), produced by the endophytic fungus *Phomopsis* sp. YM355364 from *Aconitum carmichaeli*, showed significant inhibitory activity against influenza A and moderate antifungal activities against four pathogenic fungi [219].

Phomopsterone B (**310**), an ergostane-type steroid isolated from *Phomopsis* sp. TJ507A, exhibited anti-inflammatory activity [220]. Pleurocorol A (**311**), an 11(9→8)-abeo-ergostane steroid from *Pleurotus cornucopiae*, showed inhibitory effects on nitric oxide production and cytotoxic activity against cancer cell lines [163].

Emeridones A (**312**), D (**313**), E (**314**), and F (**315**, structures in Figure 13), 3,5-demethylorsellinic-acid-based meroterpenoids from *Emericella* sp. TJ29, were isolated. Emeridone A represents the first meroterpenoid with a unique rigid 6/6/5/6 tetracyclic ring system; compounds **313** and **315** exhibited moderate cytotoxic activities [221].

Applanoids A (**316**), B (**317**), and C (**318**) with a 6/6/5/6/5 pentacyclic system, isolated from *Ganoderma applanatum*, activated the human pregnane X receptor [222]. Gloeophyllin A (**319**), an ergosteroid from *Gloeophyllum abietinum*, showed cytotoxicity against human cancer cell lines [223].

Dasyscyphins A (**320**), B (**321**), C (**322**), D (**323**), and E (**324**) from *Dasyscyphus niveus* exhibited cytotoxic activities against various human cell lines, with dasyscyphins D and E inhibiting the germination of *Magnaporthe grisea* conidia [224,225].

Ganoapplanic acids A (**325**) and B (**326**), along with ganodapplanoic acids A (**327**) and B (**328**) from *Ganoderma applanatum*, featured an uncommon C-13/C-15 oxygen bridge in a rearranged lanostane-type triterpenoid structure [4,226]. Ganolearic acid A (**329**), a rare 3/5/6/5 tetracyclic system triterpenoid, was found in *Ganoderma cochlear*, along with ganocochlearic acid A (**330**), a hexanorlanostane [227,228].

Sterenoids A–L (**331–342**) from *Stereum* sp., rare 14(13→12)-abeo-lanostane triterpenoids with 13R configurations, were isolated, with compound **336** showing potent cytotoxic activities [229]. Lanostane (**343**) was identified in *Ganoderma lucidum* [230]. These findings highlight the diversity and potential pharmacological importance of steroids and triterpenoids from various natural sources.

## 8. C-Norsteroids Derived from Plant Species

C-norsteroids, a class of steroids with a structural modification in the C ring, are represented by C23 terpenoids like apianane (**344**, structures in Figure 14) and its derivatives found in *Salvia apiana*. Derivatives such as 14-hydroxy-7-methoxy-11,16-diketo-apian-8-en-(22,6)-olide (**345**) and 7-methoxy-11,16-diketo-apian-8,14-dien-(22,6)-olide (**346**) have been identified in this plant [231]. Additionally, three other apianane terpenoids from *Salvia officinalis*, with complex structures, were isolated (**347**, **348**, and **349**) [232].

Cardenolide (**350**), with distinct antibacterial activity, was isolated from the seeds of the Cameroonian medicinal plant *Salacia staudtiana* [233]. Neoabiestrines D (**351**), E (**352**), and F (**353**) are cytotoxic rearranged lanostanes from *Abies recurvata*, with compound **352** showing potent antiproliferative effects [234]. Abiestetranes A (**354**) and B (**355**), unique tetraterpenes, were found in *Abies fabri*, demonstrating significant cytotoxic activities [234]. Lanostanes **356** and **357**, with a unique 8(14→13)-abeo-17,13-friedo moiety, were isolated from *Abies nukiangensis* [235].

Forrestiacids C (**358**) and D (**359**), rearranged spiro-lanostenes with an abietene from *Pseudotsuga forrestii*, inhibited key enzymes in the lipogenesis pathway [236]. CD-spiro-triterpenoids spirocaracolitones B (**360**), D (**361**), and **362** were identified in *Ruptiliocarpon caracolito* [237].

Officinalins A (**363**) and B (**364**), novel C23 terpenoid epimers from *Salvia officinalis*, feature a unique carbon skeleton and showed NO inhibitory activity [238]. Abibalsamins A (**365**) and B (**366**) are tetraterpenoids from *Abies balsamea*, exhibited cytotoxicity against cancer cell lines [239].

*Petilium raddeana* yielded steroidal alkaloids edpetiline (**367**) and edpetine (**368**) [240,241]. Jervine (**369**) and veratramine (**370**), and C-nor-D-homo-steroids **371–377**, were found in Veratrum species and are known for their teratogenic effects, while some veratrum alkaloids have hypotensive applications [242,243,244,245]. Fischeriana A (**378**, structures in Figure 14), a meroterpenoid from *Euphorbia fischeriana*, showed antitumor activities [246], and two C-nor-D-homo-estrones (**379** and **380**) were discovered in the Solanum family [245]. These diverse compounds highlight the potential of C-norsteroids in various therapeutic applications. 

The genus Kadsura, belonging to the Schisandraceae family and native to Asia, has been a rich source of biologically active steroids and triterpenoids, including numerous norsteroids [247]. From *Kadsura coccinea*, two triterpenoid epimers, kadcoccitones A (**381**) and B (**382**), were isolated, featuring an unprecedented 6/6/5/5-fused tetracyclic system and a C9 side chain. These compounds demonstrated anti-HIV-1 activity [248]. Neokadsuranic acid A (**383**) from *Kadsura heteroclita* and neokadsuranic acid C (**384**) from *Kadsura longipedunculata*, both with a 14(13→12)-abeo-lanostane skeleton, were identified [249,250].

Kadcoccine acids G (**385**), H (**386**), and I (**387**) from *Kadsura coccinea* showed cytotoxicity against various human tumor cell lines [251]. Kadcoccitanes A (**388**), B (**389**), and C (**390**) from the roots of the same plant, with compound **390** exhibiting anticoagulant activity, were isolated [252]. Kadcoccinones A (**391**) and B (**392**) were also detected in *Kadsura coccinea* [253].

Kadpolysperin A (**393**), a cytotoxic lanostane triterpene from *Kadsura polysperma*, demonstrated effectiveness against multiple human tumor cell lines [254]. Phyllanthoid A (**394**) from *Phyllanthus cochinchinensis* displayed moderate antifeedant activity against *Spodoptera exigua* and cytotoxicity against the MCF-7 cell line [255]. Several walsucochinoids (**395–399**) from *Walsura cochinchinensis*, with a unique carbon framework, were also isolated [256].

Ananosins A (**400**) and B (**401**) from *Kadsura ananosma*, with rearranged 5/6 consecutive carbocycle rings, were identified [257]. Kadcoccinic acids A (**402**), B (**403**), and I (**404**, structures in Figure 15) from *Kadsura coccinea*, representing 2,3-seco-6/6/5/6-fused tetracyclic triterpenoids, were isolated [258].

Neokadcoccitane A (**405**) from *Kadsura coccinea*, along with two other 14(13→12)-abeo-3,4-seco-norlanostane triterpenoids (**406** and **407**), showed moderate antiplatelet aggregation activity [259]. Longipedlactones A–I (**408–416**) from *Kadsura longipedunculata* exhibited significant cytotoxicity against various cancer cell lines [260]. Kadlongilactones A−F (**417**−**421**) from the same plant also demonstrated notable cytotoxicity [261]. These diverse compounds from Kadsura species highlight the potential of this genus in contributing to the discovery of novel pharmacologically active natural products.

A summary on the biological activity of C-norsteroids is presented in Table 3, and details are described partially in the text or a full description of the activity is written in the original articles.

## 9. D-Norsteroids

**Figure 17:**
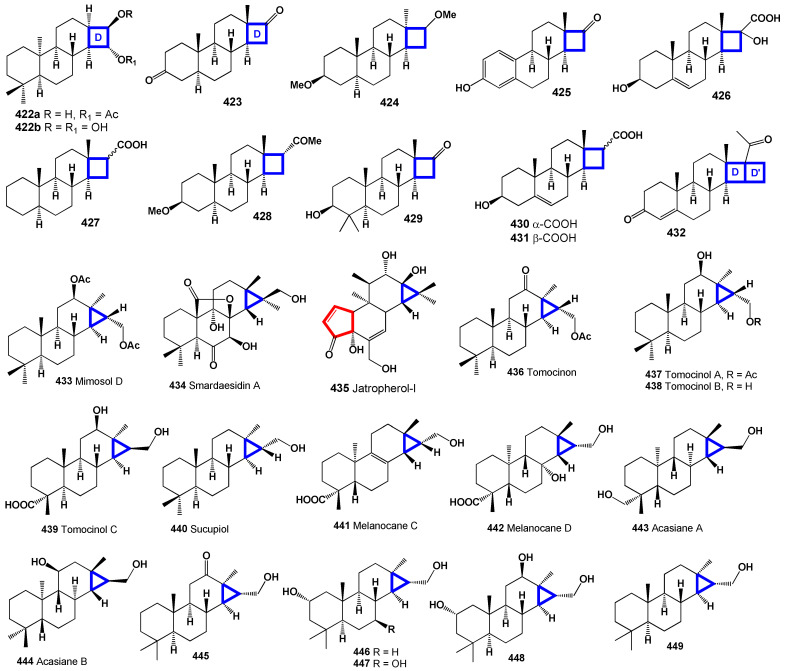
Natural and synthetic D-norsteroids.

The absence (excluding terpenoids (**422a** and **422b**, Figure 17), which are found in *Petunia patagonica* [262]) of naturally occurring classic D-norsteroids (where the D ring is decreased in size) and the existence of stable synthesized D-norsteroids are fascinating aspects of steroid chemistry [71]. This scenario can be attributed to several factors. In nature, the biosynthesis of steroids follows specific enzymatic pathways that are evolutionarily optimized for producing certain structures. These natural pathways may not favor the formation of D-norsteroids with a decreased D ring, possibly due to the structural and enzymatic constraints in the organisms that synthesize steroids. The stability of a steroid is significantly influenced by its ring structure. In general, five- and six-membered rings are more stable due to less ring strain. Decreasing the size of the D ring (for example, from five members to four) can increase the ring strain, making such structures less favorable and potentially less stable in natural conditions. In contrast to natural biosynthetic limitations, synthetic chemistry allows for greater flexibility in creating novel structures. Chemists can use various techniques to synthesize and stabilize structures that do not occur naturally, including D-norsteroids with a decreased D ring. These methods can involve using different reaction conditions, catalysts, or protecting groups that are not available in biological systems. 

The steroids naturally produced by organisms are typically those that have functional and biological significance, such as hormones like testosterone and estrogen. The absence of naturally occurring D-norsteroids with a decreased D ring suggests that such structures may not have a role or advantage in biological systems, or they could be less efficient in carrying out the necessary biological functions. The diversity of chemical structures found in nature is a result of millions of years of evolution, driven by natural selection and ecological needs. Structures that are not advantageous or are less efficient in a biological context may not be favored in natural selection, leading to their absence in natural products.

**Figure 18:**
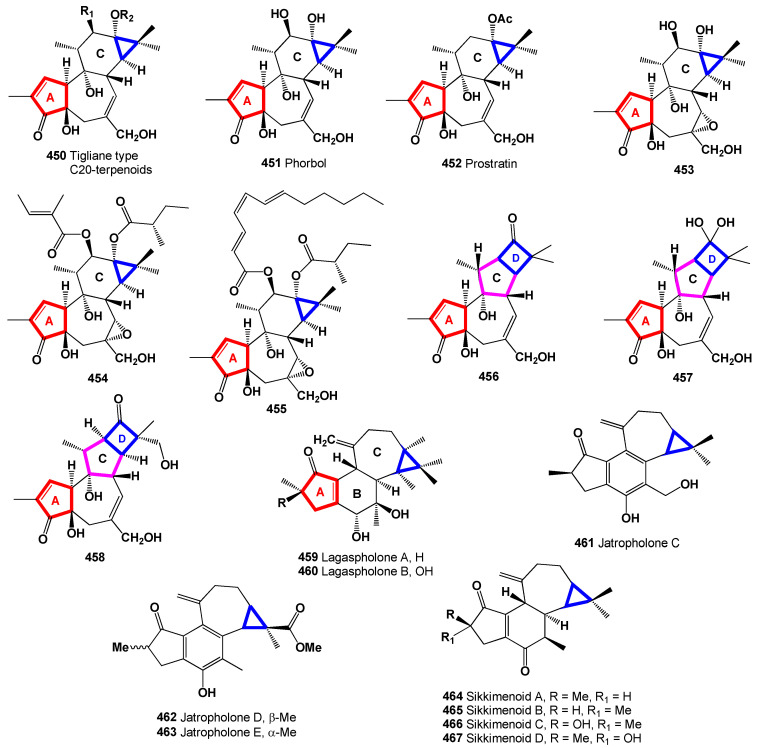
Tigliane- and jatropholane-type terpenoids derived from natural sources.

D-norsteroids are synthetic steroids distinguished by the removal of a methyl group from the carbon at position 19 of the steroid nucleus. This modification alters the molecule’s shape and properties, potentially leading to different biological activities and pharmacological profiles. D-norsteroids are researched for applications in hormone therapy, contraception, and treatment of various medical conditions [262,263,264]. The deformed D ring in the D-norsteroids (**423–432**) is highlighted in blue in Figure 17 and Figure 18.

During the 1960s and 1970s, significant research was conducted on the synthesis of D-norsteroids (**423–432**) incorporating a cyclobutane fragment, investigating their potential as hormonal drugs for reproductive system conditions, hypertension, and various cancers [265,266,267,268,269,270,271,272,273,274].

Using the norsteroid methodology, C20-terpenoids (**433–449**), isolated from various plant species, can be categorized as classical D-norsteroids. In these compounds, the D ring is reduced by two carbon atoms. Notably, while the biosynthetic pathways of typical steroids and terpenoids (**434–449**) may differ, they share a common structural framework. Mimosol D (**433**, structures in Figure 17) has been identified in extracts from *Caesalpinia bonducella* seeds and *C. mimosoides* roots. This compound exhibits significant anti-inflammatory properties, demonstrated by its ability to inhibit the production of inflammatory mediators NO and TNF-α with IC50 values of 3 μM and 6.5 μM, respectively [275]. Additionally, the isopimarane C20-terpene smardaesidin A (**434**) was isolated from the endophytic fungal strain *Smardaea* sp. AZ0432, found in the photosynthetic tissue of the moss *Ceratodon purpureus* [276].

Jatropherol-I (**435**), a phorbol-type C20-terpene, was extracted from *Jatropha curcas* seeds using ultrasonic extraction, constituting 0.04% of the seed weight. It exhibited notable insecticidal activity against several species, including *Bombyx mori*, *Lipaphis erysimi*, and *Pieris rapae*. Jatropherol-I was more effective against *B. mori* compared to *P. rapae*. After 72 h of exposure, the lethal concentration (LC_50_) for *B. mori* was 0.22 μg/mL and for *P. rapae* it was 0.83 μg/mL. Additionally, its antifeedant concentration (AFC_50_) was 0.14 μg/mL for *B. mori* and 0.57 μg/mL for *P. rapae*. This compound also demonstrated contact toxicity against aphids with an LC_50_ of 0.11 μg/insect and an AFC_50_ of 18 μg/mL for *L. erysimi*. The oral toxicity of jatropherol-I to mice was 82.2 mg/kg body weight. Its mechanism of action is believed to involve activating protein kinase C (PKC). Jatropherol-I not only activates PKC in vitro but also in vivo. In vitro studies showed a 4.99-fold increase in PKC activity in silkworm midgut cells at 100 μg/mL compared to the control. In vivo, both the activity of PKC and phosphorylation levels increased with higher dosages and prolonged exposure [277,278]. When isolated from *Jatropha* curcas oil and seed kernel, jatropherol-I was highly toxic to third instar silkworm larvae, with LC_50_ values of 0.58, 0.22, and 0.16 μg/mL at 48, 72, and 120 h, respectively. This acute toxicity correlated with changes in midgut enzyme activities and pathological alterations in midgut epithelial cells [277,278].

Three cleistanthane C20-terpenes, namely tomocinon (**436**), tomocinol A (**437**), and tomocinol B (**438**), were isolated from the ethyl acetate (EtOAc) extract of *Caesalpinia sappan* seeds. These compounds (**436–438**) represent a novel class of antiausterity agents, displaying preferential cytotoxicity against the PANC-1 human pancreatic cancer cell line under nutrient-deprived conditions, with PC_50_ values of 34.7 μM, 42.4 μM, and 39.4 μM, respectively [279]. Additionally, tomocinol C (**439**) was discovered in the seeds of the same species [280]. A pimarane-type diterpenoid, sucupiol (**440**), was isolated from *Bowdichia virgilioides* seeds, suggesting the presence of an intermediate in the biosynthesis of furanocassane-type diterpenoids [281]. Two cytotoxic diterpenoids, melanocanes C (**441**) and D (**442**), were obtained from the roots of *Aralia melanocarpa* [282], while acasiane A (**443**) and acasiane B (**444**) were isolated from *Acacia farnesiana* roots [283]. Several other diterpenoids (**445–449**) were found in extracts from seeds, roots, leaves, or bark of trees belonging to the Euphorbiaceae, Fabaceae, Leguminosae, and Rosaceae families [284,285,286,287,288,289].

Another interesting group of terpenoids, which can be classified as classic D-norsteroids, includes those containing the gem-dimethylcyclopropyl unit, particularly the tigliane and jatropholane types. These metabolites synthesized by plants share a common intermediate, casbene, formed by cyclizing geranylgeranyl pyrophosphate and ultimately retaining the cyclopropane ring [290,291,292,293]. The literature indicates two types of C20-terpenoids, tigliane (**450**, structures in Figure 18) and jatropholane, which can be considered D-norsteroids.

Tigliane C20-terpenoids are predominantly found in the Thymelaeaceae and Euphorbiaceae plant families. Their structural diversity stems from the presence of polyoxygenated functionalities within their polycyclic skeletons. These diterpenoids are known for their toxicity, yet they have demonstrated a range of biological activities, including anticancer, anti-HIV, and analgesic effects, making them significant in the field of natural product drug discovery. Tigliane diterpenoids feature a 5/7/6/3 (A/B/C/D)-fused tetracyclic structure, with the D ring forming a gem-dimethylcyclopropane ring. In tiglianes isolated from Thymelaeaceae plants, the A/B and B/C rings are trans-fused, while the C/D ring is cis-fused. A notable compound within this group is phorbol (**451**), characterized by an α,β-unsaturated ketone in the A ring, a C-6 to C-7 double bond, a primary hydroxy group at C-20, a secondary hydroxy group at C-12, and tertiary hydroxy groups at C-4, C-9, and C-13. Phorbol esters, the most canonical class of tiglianes, are derivatives where the hydroxy groups at C-12, C-13, or C-20 of phorbol are esterified. Additionally, there is 12-deoxytigliane, which lacks a substituent at C-12. While most known tiglianes are from Euphorbiaceae plants, those from Thymelaeaceae exhibit diverse oxidative modifications on the B ring, highlighting their structural variety [294,295,296,297].

Phorbol (**451**) was first identified in 1934 as a hydrolysis product of croton oil, derived from the seeds of *Croton tiglium*, the purging croton [298]. Another protein kinase C activator, prostratin (**452**), was discovered in Euphorbia species [299].

The Australian blushwood tree, *Fontainea picrosperma*, is known for its kernels that yield veterinary anticancer drugs like phorbol (**453**), 5β-hydroxy-6α,7α-epoxyphorbol (**454**), and phorbol diesters (**455**) [300]. Recently, Huang and colleagues [301] discovered two new tigliane diterpenoids through the hydrolysis of phorbol: 4β,9α,20-trihydroxy-14(13→12)-abeo-12βH-1,6-tigliadiene-3,13-dione (**456**) and 4β,9α,13,13,20-pentahydroxy-14(13→12)-abeo-12βH-1,6-tigliadiene-3-one (**457**). These compounds feature a cyclobutane D ring. Furthermore, chemical analysis of the roots of *Euphorbia ebracteolata* yielded a rare 14(13→12)-abeo-tigliane diterpenoid (**458**) [302], a unique case of a C20-steroid with a cyclobutane ring, indicating the potential discovery of D-norsteroids in the future.

Additionally, tigliane diterpenoids form a substantial group of biologically active metabolites, known for their anti-HIV-1 properties and activation of protein kinase C [303,304,305]. Two diterpenes, lagaspholones A (**459**) and B (**460**), were isolated from the methanol ice extract of *Euphorbia lagascae*. Both compounds exhibit a rare jatropholane-type skeleton with a 5:6:7:3-fused-ring system [306]. Three jatropholane-type diterpenoids, jatropholones C (**461**), D (**462**), and E (**463**), were isolated from the roots of *Jatropha curcas* [307]. Similar C20-terpenoids, named sikkimenoids A–D (**464–467**), were found in the extracts of the aerial parts of *Euphorbia sikkimensis* [308].

Nature, compensating for the absence of classic D-norsteroids in living organisms, offered variants of pseudo D-norsteroids by removing the D ring at a distance of one carbon–carbon (C-C) bond (for example, strophasterols (**468–473**) and/or tricholumin A (**474**)). The carbon–carbon (C-C) bond distances in steroids, like in most organic molecules, can vary slightly depending on the specific structure and environment of the molecule. In alkanes, which are the simplest type of hydrocarbon chains with single bonds, the average C-C bond length is typically about 1.54 Å (angstroms). Since steroids are largely composed of carbon rings that involve single bonds, their C-C single bond distances are expected to be in this range as well. Such examples can be natural steroids (**468–491**) in which the D ring is removed at various distances from the smaller C ring.

**Figure 19:**
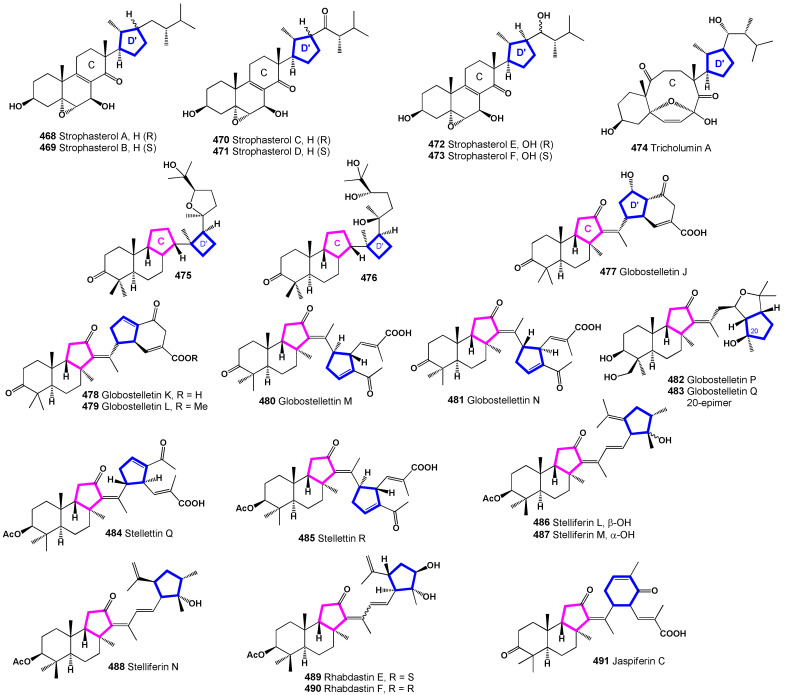
Natural terpenoids with D ring removed.

Strophasterols (**468–473**, structures in Figure 19), natural D-norsteroids from the mushroom *Stropharia rugosoannulata*, lack the D ring and form a remote D’ ring through a “retro-aldol” reaction [309,310]. Strophasterols E and F (**472** and **473**) were isolated from *Pleurotus eryngii*, showing interesting structural properties [311]. Strophasterol C (**471**) and glaucoposterol A (**472**) were obtained from *Cortinarius glaucopus* [312]. Tricholumin A (**474**), an ergosterol derivative from *Trichoderma asperellum*, exhibited antimicrobial activity [313].

Unusual malabaricane triterpenes (**475** and **476**) with a cyclobutane ring were isolated from *Ailanthus malabarica* [314]. In marine pharmacology, globostelletins (**477–483**) from *Rhabdastrella globostellata* and stellettins Q and R (**484** and **485**) from *Stelletta* sp. are examples of D-norsteroids with cyclopentane units linked to different positions of sidechains [315,316]. Stelliferins L (**486**), M (**487**), and N (**488**) from *Rhabdastrella* cf. *globostellata* exhibited antimicrobial activity [317]. Rhabdastins E and F (**489** and **490**) from *Rhabdastrella globostellata* showed weak activity [318]. Jaspiferin C (**491**), an isomalabaricane-type triterpenoid from *Jaspis stellifera*, possesses a unique six-membered carbon ring [319]. These studies highlight the diversity and potential of D-norsteroids in drug discovery and development.

A summary on the biological activity of D-norsteroids is presented in Table 4, and details are described partially in the text or a full description of the activity is written in the original articles.

## 10. Modified D Ring in Steroids

The term ‘D ring modified’ in steroid chemistry denotes an alteration to the D ring of the steroid nucleus. This modification might include variations in the ring’s shape, angular structure, or bond lengths, deviating from the typical cyclopentane ring structure common in most steroids. Such changes could result from chemical reactions, physical forces, or interactions with other molecules. Altering the D ring can markedly influence the biological activity and characteristics of the steroid [71]. Steroids are used in a variety of biological contexts, including as hormones and in medications. Changes in the ring structure can alter how these compounds interact with biological systems, potentially changing their effectiveness or function [71,320,321].

According to environmental and/or physiological principles, nature is unable to synthesize classical D-norsteroids, i.e., four-membered D rings, however, she came up with a mechanism that de facto provides such an opportunity. For example, by removing a carbon atom from the D ring and introducing an oxygen atom in its place, we obtain a new stroid construct with a tetrahydrofuran D ring, and this construct is called a meroterpenoid.

The conformation and reactivity of steroids with a cyclobutane D ring or a tetrahydrofuran D ring differ significantly from those of typical steroids with a cyclopentane D ring. These modifications lead to unique structural and chemical properties [71,320,321].

Cyclobutane is a four-membered ring, which is significantly more strained than the typical five-membered D ring in steroids. This strain arises from the angle strain and torsional strain due to the smaller ring size, which forces the carbon atoms into a less favorable alignment compared to a cyclopentane ring. The strain in the cyclobutene ring makes these steroids more reactive. The ring is more prone to chemical reactions, such as ring-opening reactions, due to the high-energy conformation. This could potentially be exploited in drug design, where the ring could be a site for targeted modification or activation.

**Figure 20:**
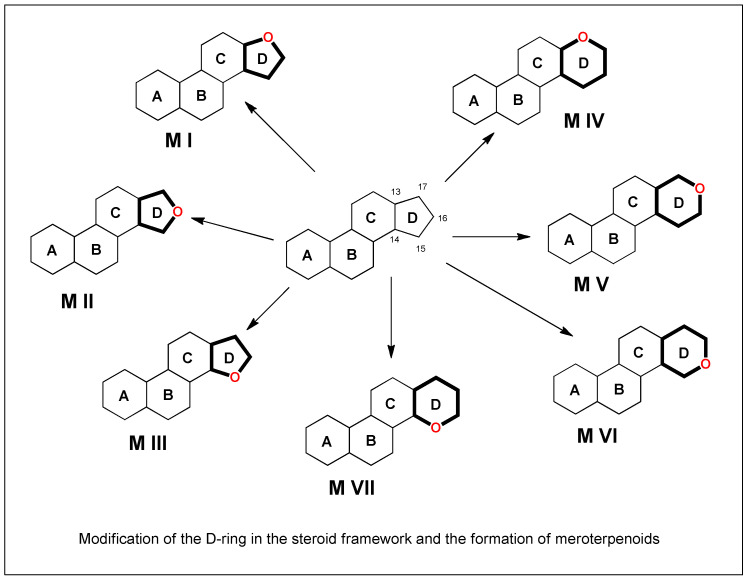
Natural meroterpenoids are formed by modification of the D ring in steroid hormones. Oxygen is marked in red.

A tetrahydrofuran is a five-membered ring containing an oxygen atom. Replacing the carbon-only ring with an oxygen-containing ring changes the electronic properties of the ring. The presence of oxygen introduces heteroatom characteristics, like different electronegativity and bond angles, altering the 3D conformation of the ring (see Figure 20). The presence of oxygen in the ring affects the reactivity of the steroid. The electron-rich oxygen can participate in hydrogen bonding and other interactions, potentially affecting how the steroid interacts with biological molecules. It can also influence the stability and reactivity of adjacent functional groups. Both types of modifications (cyclobutane and tetrahydrofuran D rings) can significantly alter the biological activity of the steroid. Such changes can impact the binding affinity of the steroid to receptors, its metabolic stability, and its overall pharmacological profile. This kind of structural modification is a key area in medicinal chemistry for designing new drugs with enhanced properties or reduced side effects. 

Meroterpenoids with a tetrahydrofuran D ring have been found in nature, and this suggests that this structure is more stable than D-norsteroids [322,323,324]. When the D ring is deformed in steroid hormones, there are seven different variations of natural meroterpenoids as shown in Figure 20 and they depend on the position of oxygen in the D ring. 

**Figure 21:**
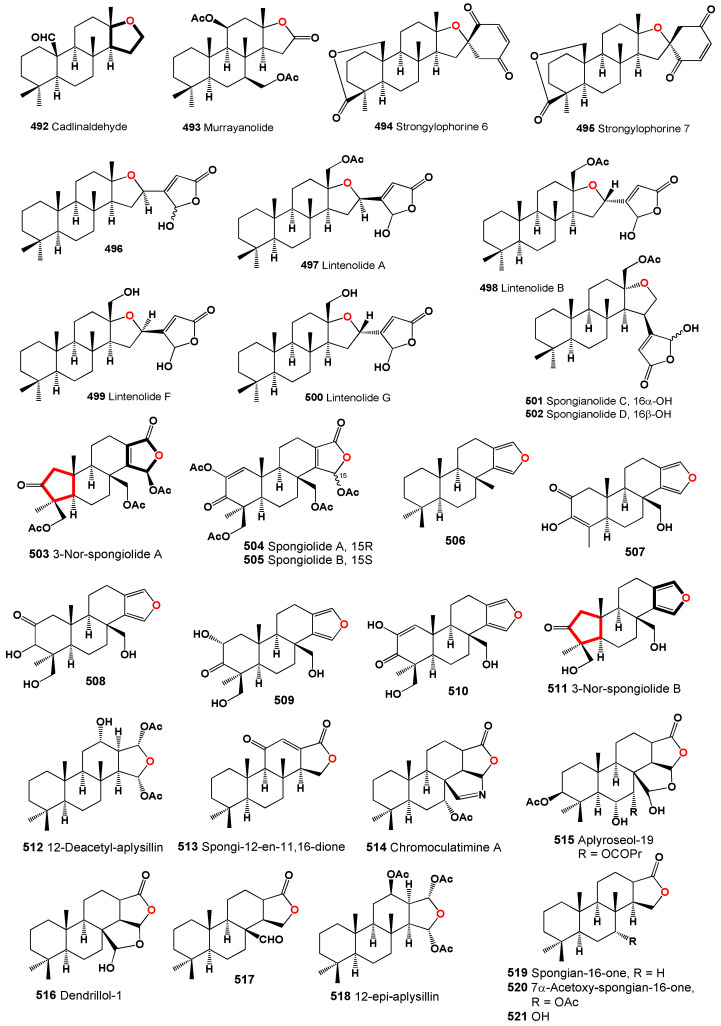
Meroterpenoids derived from natural sources.

Marine invertebrates have yielded several metabolites with an oxygen atom at position 17 (group M I, see Figure 21). A terpenoid, cadlinaldehyde (**492**, structures in Figure 20), with a unique degraded sesterterpenoid skeleton, was isolated from the skin extracts and egg masses of the northeastern Pacific dorid nudibranch *Cadlina luteomarginata* [325]. 

A distinctive C21 tetracyclic terpenoid lactone, murrayanolide (**493**), was sourced from the marine bryozoan *Dendrobeania murrayana* [326]. Strongylophorines 6 (**494**) and 7 (**495**) were identified in extracts from the sponge *Strongylophora durissima*, found near Maricaban Island in the Philippines [327]. A cheilanthane sesterterpenoid, 13,16-epoxy25-hydroxy-17-cheilanthen-19,25-olide (**496**), acting as a protein kinase inhibitor, was isolated from the marine sponge *Ircinia* sp. [328].

Pentacyclic sesterterpenes, lintenolide A (**497**) and lintenolide B (**498**), were characterized from the Caribbean sponge *Cacospongia* cf. *linteiformis*. Both compounds exhibited high ichthyotoxicity and antifeedant activity, suggesting their role as natural feeding deterrents [329]. Ichthyotoxicity tests on *Gambusia affinis*, the mosquito fish, revealed that lintenolides A and B are toxic at a concentration of 10 ppm. Antifeedant assays with *Carassium auratus*, the goldfish, indicated strong feeding deterrence at a concentration of 30 μg/cm^2^ of food pellets. Furthermore, lintenolides A and B inhibited protein kinase C (PKC) at IC_50_ values of 20–30 μg/mL and did not inhibit human 85 kD phospholipase A2 (PLA2). They also potently inhibited (IC_50_ 0.50–1.40 μg/mL) the proliferation of the MCF-7 mammary tumor cell line [330]. Two more compounds, lintenolides F (**499**) and G (**500**), were identified as antiproliferative sesterterpenes from the same marine sponge [331].

The marine sponge *Spongia* sp. has been identified as a source of tricyclic sesterterpenoids, spongianolides C (**501**) and D (**502**). These compounds, featuring a γ-HB moiety, have been found to inhibit the proliferation of the MCF-7.4 mammary tumor cell line [330].

**Figure 22:**
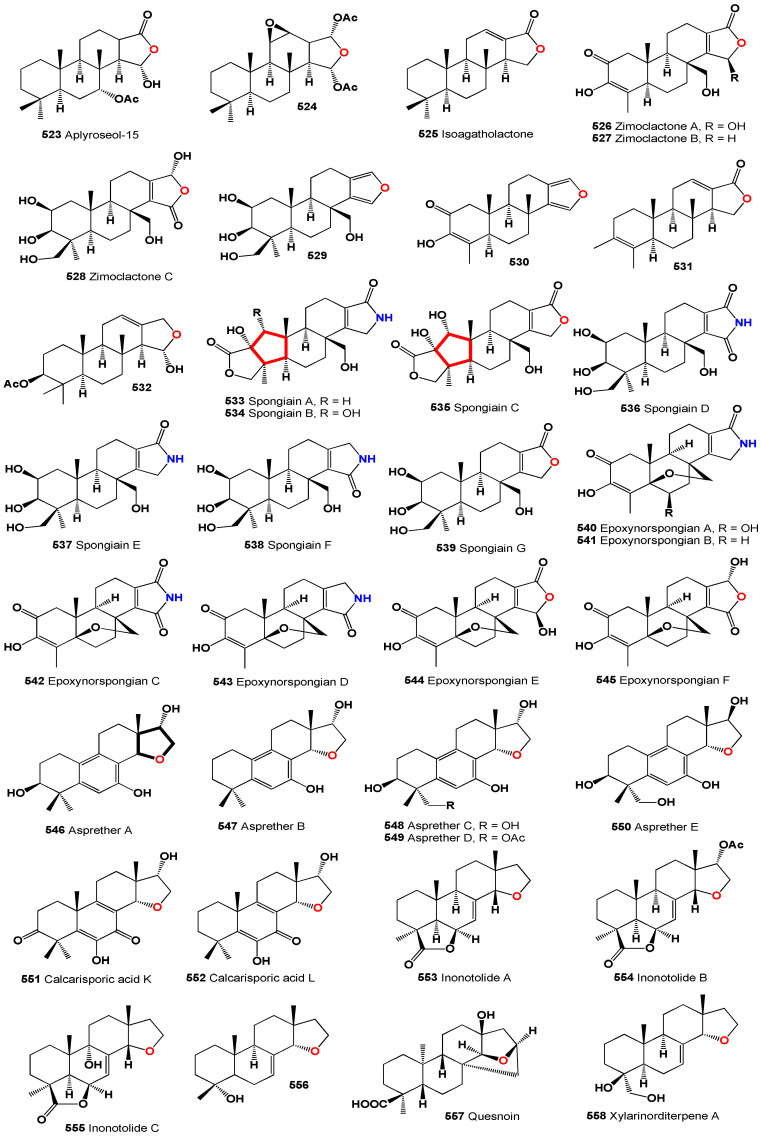
Meroterpenoids derived from marine organisms and fungi.

A significant diversity of meroterpenoids has been discovered in marine invertebrates, particularly in those where oxygen (or another heteroatom, such as nitrogen) is located at position 16 of the D ring (group M II, see Figure 22). The South China Sea sponge *Spongia officinalis* yielded 3-nor-spongiolide A (**503**), a rare 3-nor-spongian carbon skeleton, and spongiolides A (**504**) and B (**505**), which uniquely feature a γ-butenolide in place of the typical furan ring in the D ring. Alongside these, six related metabolites (**506–511**) were also isolated as its metabolic components [332].

Numerous oxygenated meroterpenes (**512–517**) have been discovered in the Australian nudibranch *Chromodoris reticulata* [333]. 12-*epi*-Aplysillin (**518**) and another compound (**522**) were detected in extracts from the nudibranch *Chromodoris luteorosea* [334], while spongian-16-one (**519**) and its 7α-acetoxy derivative (**520**) were found in *Chromodoris inopinata* [335]. These latter two compounds, **519** and **520**, were also reported by Miyamoto and colleagues [336] from the Japanese chromodorid *Chromodoris obsoleta*.

A minor metabolite, aplyroseol-15 (**523**, structures in Figure 21), was isolated from the marine sponge *Aplysilla rosea* [337]. An anticancer metabolite containing an epoxy group (**524**) was isolated from the mollusk *Chromodoris obsoleta*, exhibiting strong cytotoxicity against L1210 and KB cancer cells [338]. Isoagatholactone (**525**) was obtained from the sponge *Spongia officinalis* [339].

Recent studies have identified bioactive metabolites such as zimoclactones A (**526**), B (**527**), and C (**528**) from the marine sponge *Spongia zimocca* sp. *irregularia* [340,341]. Zimoclactone A demonstrated moderate cytotoxic activity against P388 cell lines [340,341]. Two spongian C20-terpenoids, **529** and **530**, were derived from a Great Barrier Reef sponge, *Spongia* sp. [342]. Additionally, two oxidized diterpenoids, 3-methylspongia-3,12-dien-16-one (**531**) and 3β-acetoxy-15-hydroxyspongia-12-en (**532**), were isolated from the marine sponge *Acanthodendrilla* sp., collected in Pulau-Pulau [343].

Spongiains A–C (**533**–**535**), the first examples of spongian diterpenes with a pentacyclic skeleton composed of a fused 5/5/6/6/5-ring system through ring A rearrangement, and four new spongian diterpenes, spongiains D–G (**536**–**539**), were isolated from the marine sponge *Spongia* sp. [344]. Novel epoxynorspongians A–F (**540–545**), new 19-norspongian diterpenes with a 5,17-epoxy unit, were also isolated from *Spongia* sp. Compound **544** displayed moderate activities against the PC3 and PBL-2H3 cell lines, with IC_50_ values of 24.8 and 27.2 μM, respectively [345].

**Figure 23:**
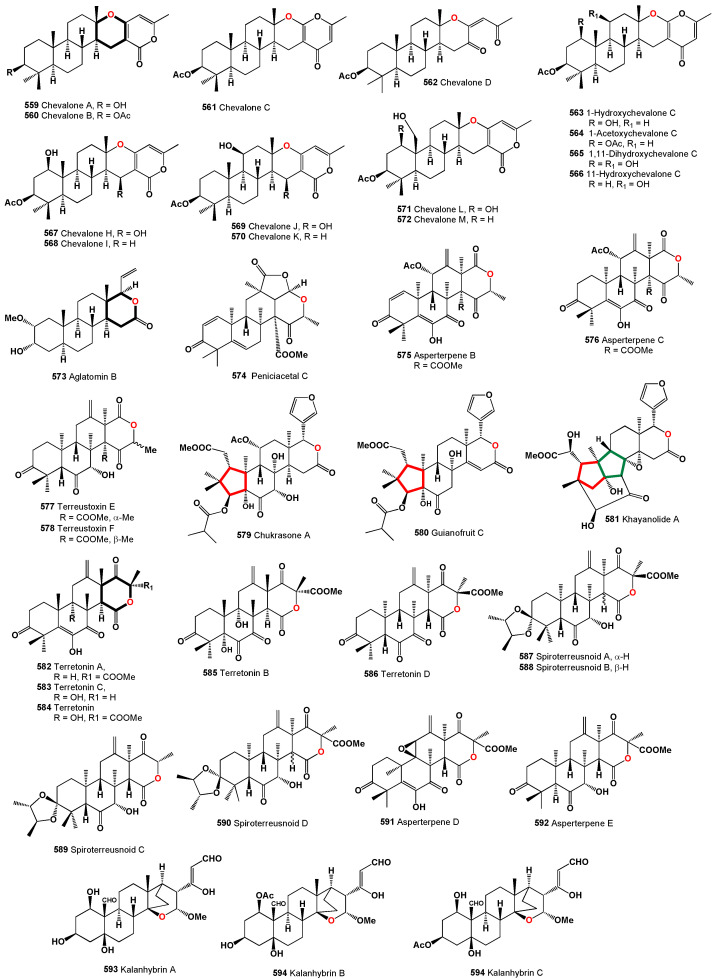
Meroterpenoids derived from fungal endophytes, fungi, and plants.

Several metabolites with an oxygen atom at position 15 (group M III, see Figure 23) have been discovered in fungi and plants. From *Aspergillus wentii*, meroterpenoids named asprethers A–E (**546–550**) were isolated and tested for cytotoxicity. These compounds showed effectiveness against the A549 cell line, with IC_50_ values of 20, 16, 19, 17, and 20 μM, respectively. Specifically, compound **546** exhibited higher activity against the T-47D cell line, while compound 547 was more effective against the HEK293 and SMMC-7721 cell lines [346].

The fungus *Calcarisporium arbuscula* produces calcarisporic acids K (**551**) and L (**552**) [347]. Inonotolides A–C (**553**–**555**), isolated from the fungus *Inonotus sinensis*, represent another group of compounds [348]. From the endophytic fungus *Xylaria* sp., the isopimarane diterpene 14α,16-epoxy-18-norisopimar-7-en-4α-ol (**556**), exhibiting moderate antifungal activity, was obtained [349].

Fossilized tree resin, amber, found near the Oise River in the Paris basin, France, and dated to be 55 million years old, yielded the novel meroterpenoid quesnoin (**557**). The absolute configurations of its eight chiral centers were determined as 4S, 5S, 8R, 9S, 10S, 13S, 14R, and 16S. Quesnoin revealed the tree producer to be akin to modern *Hymenaea oblongifolia*, Fabaceae, a subfamily of Caesalpiniaceae, one of the oldest angiosperms. The discovery of *H. oblongifolia* suggests the Paris basin may have had a tropical climate in the early Eocene period, 55 million years ago [350]. Nor-isopimarane diterpene, xylarinorditerpene A (**558**), was isolated from the fungicolous fungus *Xylaria longipes* HFG1018, isolated from *Fomitopsis betulinus* [351].

The modified D ring in steroids, as shown in Figure 23, is categorized into four groups based on the position of the oxygen atom in the six-membered ring. Representatives of these groups (M IV–M VII) of meroterpenoids have been extracted from marine and terrestrial sources. Meroterpenoids named chevalones A–D (**559**–**562**, structures in Figure 22) were isolated from the fungus *Eurotium chevalieri*. Compound **562** exhibited antimalarial activity against *Plasmodium falciparum*, and **561** showed antimycobacterial activity against *Mycobacterium tuberculosis*. 

Compounds **560**–**562** also displayed cytotoxicity against cancer cell lines [352]. Chevalone C analogs, 1-hydroxychevalone C (**563**), 1-acetoxychevalone C (**564**), 1,11-dihydroxychevalone C (**565**), and 11-hydroxychevalone C (**566**), were isolated from the fungus *Neosartorya spinosa*. 1-hydroxychevalone C showed antimycobacterial activity against *Mycobacterium tuberculosis* with a MIC value of 26.4 μM, and 1-acetoxychevalone C exhibited antimalarial activity against *Plasmodium falciparum* with an IC_50_ value of 6.67 μM. These compounds also demonstrated cytotoxicity against KB and NCI-H187 cancer cell lines, with IC_50_ values ranging from 32.7 to 103.1 μΜ [353]. New α-pyrone meroterpenoid chevalones H–M (**567**–**572**) were isolated from the gorgonian-coral-derived fungus *Aspergillus hiratsukae* SCSIO 7S2001 collected from Mischief Reef in the South China Sea. All compounds displayed various degrees of antibacterial activity, with MIC values between 6.25–100 µg/mL [354].

Meroterpenoid aglatomin B (**573**) was isolated from the leaves of *Aglaia tomentosa* and found in the bark of *Aglaia lawii* [355]. A highly oxygenated meroterpenoid, peniciacetal C (**574**), with a unique 3,6-dimethyldihydro-4H-furo[2,3-b]pyran-2,5-dione unit and a 6/6/6/5/6-fused pentacyclic skeleton, was detected in extracts from the mangrove-derived fungus *Penicillium* sp. HLLG-122 [356].

Aspermeroterpenes B (**575**) and C (**576**) were obtained from the marine-derived fungus *Aspergillus terreus* GZU-31-1, isolated from the air-breathing sea slug *Onchidium struma* [357]. Highly oxygenated meroterpenoids, terreustoxins E (**577**) and F (**578**), were isolated from the Antarctic fungus *Aspergillus terreus* [358].

Chukrasone A (**579**), incorporating a highly rearranged A/B-ring system, was isolated from *Chukrasia tabularis* and exhibited potential inhibition of the delayed rectifier (IK) K+ current [359]. A chukrasone-type limonoid, guianofruit C (**580**), was found in the fruit oil of *Carapa guianensis* (Meliaceae), a traditional medicine in Brazil and Latin American countries, and showed moderate inhibitory activities [360]. Khayanolide A (**581**), a rearranged phragmalin-type limonoid with an A, B, D-seco compound structure, was isolated from the ether extract of the stem bark of *Khaya senegalensis* as an insect antifeedant. Khayanolide A demonstrated antifeedant activity against the third instar larvae of *S. littoralis* [361].

Several sesterterpenes have been reported in *Aspergillus terreus*, such as terretonins A (**582**), B (**585**), C (**583**), D (**586**), and terretonin (**584**) [362]. Endophytic *Aspergillus terreus*, associated with the root of *Tripterygium wilfordii* (Celastraceae), yielded spiro-dioxolane-containing adducts with 3,5-DMOA-based meroterpenoid and 2,3-butanediol moieties, named spiroterre usnoids A–D (**587**–**590**) [363].

Asperterpenes D (**591**) and E (**592**) have been obtained from extracts of the soil-derived *Aspergillus terreus* [364]. Furthermore, purification of the cytotoxic fractions of the methanol extracts of the succulent plant *Kalanchoe hybrida* leads to the isolation of three compounds characterized by the basic skeleton of α-pyrone ring-opening products of bufadienolides, namely, kalanhybrins A–C (**593**–**595**) [365].

In summary, this text highlights natural meroterpenoids (**492–594**) that are formed by the modification of the D ring in steroids. The meroterpenoids discussed are primarily formed through the oxidation of corresponding steroids. However, there are notable exceptions: compounds **533**, **534**, **537**, **538**, **540**, **541**, **542**, and **543**, which uniquely contain nitrogen instead of oxygen as a heteroatom. This demonstrates the diversity in the biosynthesis of the compounds presented, emphasizing the variability in terpenoid structures. Additionally, we provide literature on the synthesis of norsteroids [366,367,368,369,370,371,372] and their biological activity [373,374,375,376,377,378].

A summary on the biological activity of meroterpenoids is presented in Table 5, and details are described partially in the text or a full description of the activity is written in the original articles.

## 11. Synthesis of Norsteroids

The 1930s were a pivotal decade in the study of natural steroids and their chemical synthesis [379,380,381,382,383,384,385,386]. This era is often referred to by steroid chemists as the “decade of the sex hormones,” a period marked by the determination of the molecular structures of certain sex hormones and their introduction into medical practice as drugs. Russell Marker achieved a significant milestone during this time with the first practical synthesis of the pregnancy hormone, progesterone, using a process now known as the Marker degradation. He produced progesterone from a starting material found in a species of Mexican yam, and this progesterone eventually became the preferred precursor in the industrial preparation of the anti-inflammatory drug cortisone. Important research on sex hormones continued in Mexico, leading to the synthesis of the first useful oral contraceptive in 1951 [384,385,386,387,388,389,390,391,392].

**Scheme 1:**
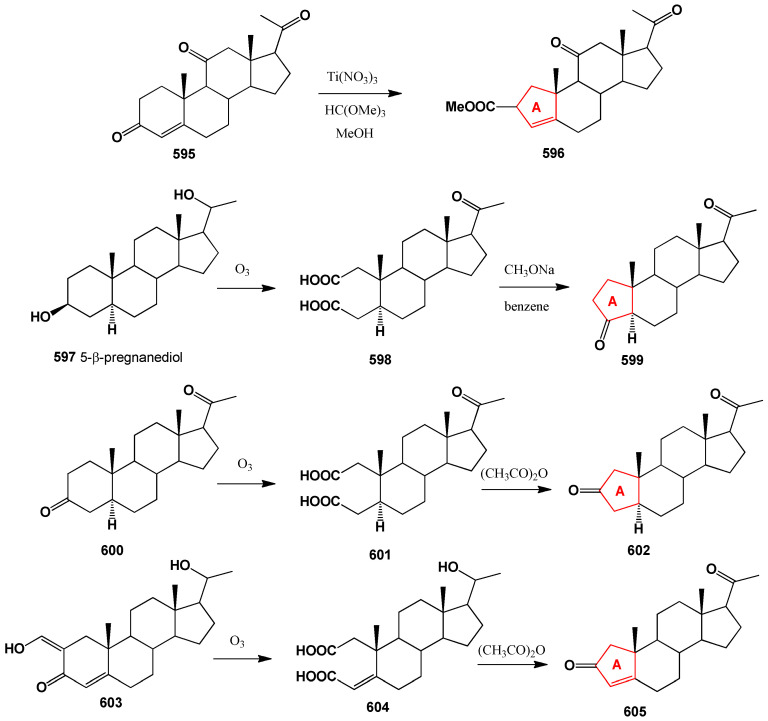
Synthesis of A-norsteroids.

The ring contraction reaction of 11-ketoprogesterone (**595**, see Scheme 1) was successfully carried out on a large scale. The purity of the product and its structure as 11-keto A-norsteroid (**596**) were confirmed by analytical and spectroscopic (IR, NMR, MS) methods. The method developed in Thomas’ laboratory was repeated on a larger scale, carrying out the ring contraction of 11-ketoprogesterone using thallium nitrate in a mixture of trimethyl orthoformate and methanol to give an A-norsteroid (**596**) [393].

The first A-norpregnane was prepared by Butenandt [394]. They oxidized 5β-pregnanediol (**597**) to the 20-oxo-3,4-seco acid derivative (**598**). Upon heating with acetic anhydride, the A ring closed with the evolution of water and carbon dioxide to give A-nor-5β-pregnan-3,20-dione (**599**). Because the 5β-pregnan series has an A/B-cis-ring junction, enolization of the 3-oxo derivative will be directed toward the C4 position, and oxidative cleavage will give 3,4-seco acid. Using a similar approach, Marker and co-workers [388,392] oxidized 5α-pregnane-3,20-dione (**600**) to 2,3-seco acid (**601**). Treatment with acetic anhydride gave A-nor-5α-pregnan-2,20-dione (**602**).

Weisenborn and Applegate synthesized A-norprogesterone [379]. 2-Hydroxymethylenepregn-4-en-20β-ol-3-one (**603**) was cleaved with ozone to give the unsaturated 2,3-seco acid (**604**). Treatment with acetic anhydride gave A-nor-α,β-unsaturated ketone, and mild oxidation gave A-norprogesterone (**605**).

**Scheme 2:**
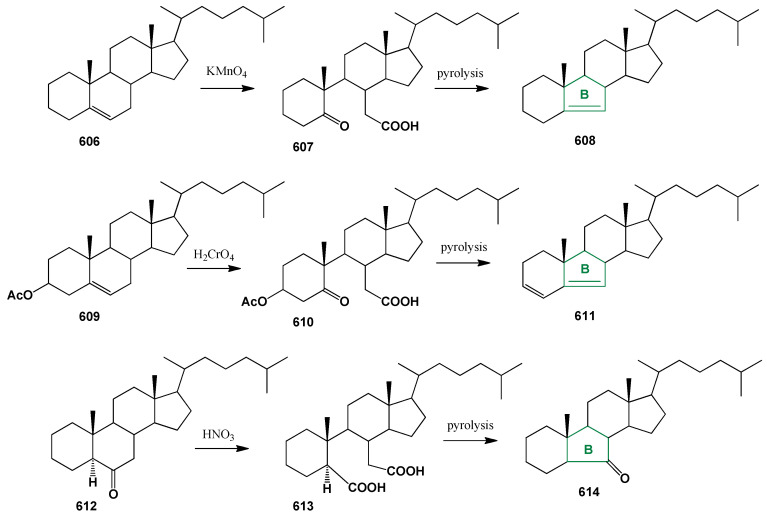
Synthesis of B-norsteroids.

The first B-norsteroids were obtained by cleaving the Δ^5^-double bond of cholesterol derivatives, followed by cyclization of the resulting keto-acids (see Scheme 2). Windaus prepared Δ^5^-B-norcholestene (**608**) by permanganate oxidation of Δ5-cholestene (**606**) and pyrolysis of a keto-acid (**607**). Similarly, Windaus prepared Δ^3,5^-B-norcholestene (**611**) by pyrolysis of a resinous keto-acid (**610**) obtained in low yield from the chromic acid oxidation of cholesteryl acetate (**609**). It was later shown that 6,7-seco-diacid (**613**), obtained from nitric acid oxidation of cholestane-6-one (**612**), could be cyclized to a B-nor-ketone (**614**) by pyrolysis of barium salt [395].

**Scheme 3:**
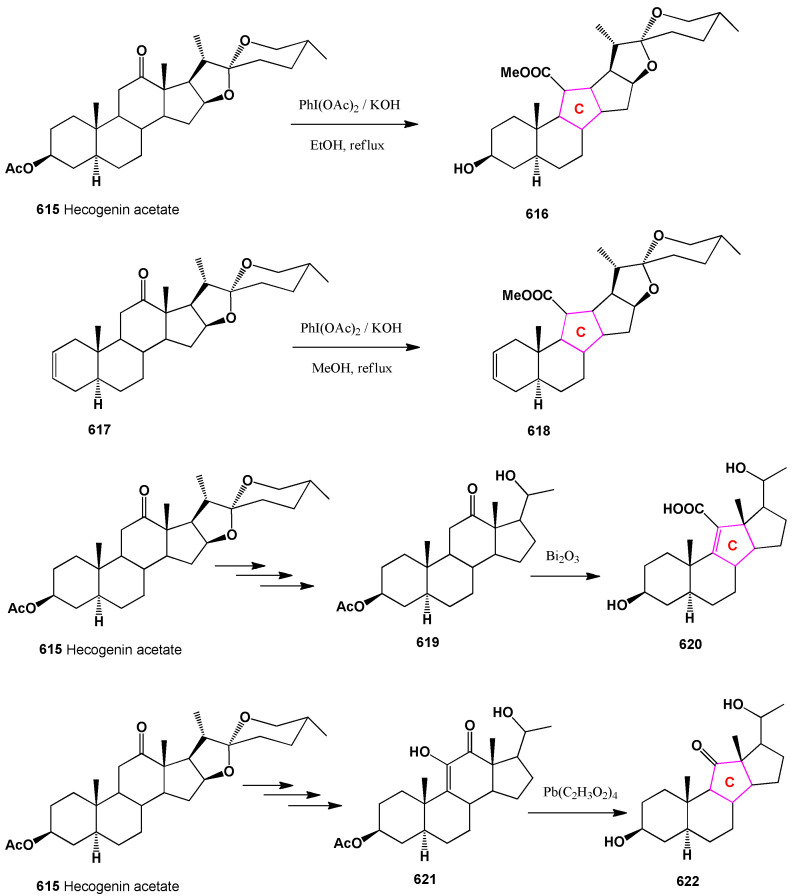
Synthesis of C-norsteroids.

When the commercially available hecogenin acetate (**615**, see Scheme 3) was refluxed in ethanol with different amounts of PhI(OAc)2 and KOH, variable yields of the C-ring contraction product **616** were obtained. The best result (58%) was achieved when hecogenin acetate was refluxed for 24 h with PhI(OAc)2 and KOH. In all cases, variable amounts of hecogenin (product of the hydrolysis of the acetate in C-3) were obtained. All attempts at improving the yield of the C-norsteroid **616** by either extending the reaction time or increasing the amount of reagent resulted in lower yields and products of deteriorated purity [396].

A steroidal ketone (**617**), PhI(OAc)_2_, and KOH in methanol were refluxed for 24 h before pouring into a cold saturated NaCl solution. The resulting solid was filtered and washed with water. Chromatographic purification on silica gel employing a gradient of hexane to an 85/15 hexane/ethyl acetate mixture as eluent afforded a rearranged product (**618**) [396].

Hecogenin acetate (**615**) is a readily available substance for the preparation of steroids with a modified C ring, and it was converted to 3β,20-diacetoxy-5α-pregnan-12-one (**619**). The 11,12-ketol group in **619** was oxidized using bismuth trioxide to form a C-norsteroid (**620**). Another use of hecogenin acetate was conversion to 3β,20-diacetoxy-11-hydroxy-preg-9-en-12-one (**621**). Treatment of this compound with lead tetra-acetate gives the final product 3β,20-dihydroxy-C-nor-pregnan-11-one (**622**) [397].

**Scheme 4:**
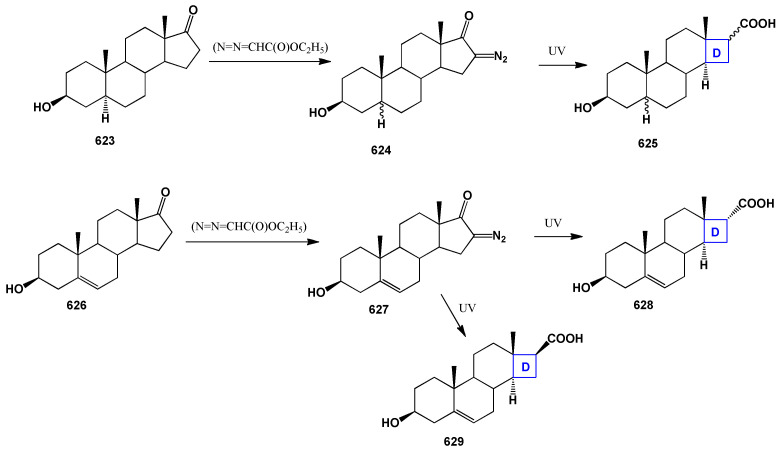
Synthesis of D-norsteroids.

The preparation of 16-diazo-androstan-3β-ol-17-one (**624**) from 5α-androstan-3β-ol-17-one (**623**) has been described (see Scheme 4). The 16-diazo group has been replaced by 16β-acetate, 16α-halogen, and 16ζ-alkoxy substituents. Irradiation of the 16-diazo compound by UV light leads to contraction of the D ring in the steroid molecule, resulting in the formation of a D-norsteroid (**625**) [268].

Starting with androst-5-en-3β-ol-17-one (**626**), the corresponding androst-5-en-16-diazo-17-one (**627**) was synthesized. Irradiation of this diazo compound caused contraction of the D ring, leading to the production of D-nor carboxylic acids (**628** and **629**) [272,398].

We have presented some syntheses of norsteroids, and that those steroids containing the diazo group under the influence of UV light form D-norsteroids, which are not found in nature, is interesting.

## 12. Conclusions

In conclusion, the exploration of A, B, C, and D-norsteroids presents a fascinating and fruitful area in the field of steroid chemistry and pharmacology. These compounds, characterized by their unique structural modifications from the typical four-ring steroid structure, exhibit a wide range of biological activities and potential therapeutic applications. The removal or alteration of specific methyl groups or changes in the steroid nucleus results in unprecedented molecular architectures, which in turn influence their interactions with biological systems.

The significance of these steroids is underscored by their diverse origins, from natural sources like marine organisms to synthetic laboratory creations. Marine sponges, corals, mollusks, fungi, and other organisms have been highlighted as rich sources of these unique compounds. Their varied ecological niches foster the production of steroids with novel structures, often with significant biological activity, such as cytotoxic, antimicrobial, and anti-inflammatory properties. These findings are not only crucial for understanding the ecological roles of these compounds but also for their potential in drug discovery and development.

The therapeutic potential of A, B, C, and D-norsteroids is vast, with applications ranging from hormone therapy and contraception to the treatment of various cancers and inflammatory diseases. The historical interest in these compounds, particularly in the context of hormonal drug development, has set the stage for ongoing research and exploration.

The article concludes by emphasizing the importance of continued research in this field. The unique structural features and biological activities of A, B, C, and D-norsteroids hold great promise for the development of novel pharmacological agents. Future studies are essential for unraveling their full potential, understanding their mechanisms of action, and harnessing their capabilities for medical advancements. In essence, the world of modified steroids offers a treasure trove of possibilities for advancing human health and medicine.

## Figures and Tables

**Table 1:** Summarized biological activity of A-norsteroids.

Steroid No.	Reported Activity of A-Norsteroids	Ref.
**52–55**	Moderate cytotoxicity against HCT-116 tumor cells	[47]
**61–63**	Antifouling properties	[53]
**66**	Calcium-binding activity	[55]
**80,81**	Inhibitory effects on membrane-type matrix metalloproteinase	[62]
**105**	Strong cytotoxic activity against human cancer cell lines HL-60 and PC-3	[74]
**106**	Antibacterial activity against Gram-positive bacteria	[75]
**107**	Antiacetylcholinesterase activity Strong cytotoxicity against PACA, A431, and HepG2 cells	[76,77]
**108–110**	Resistance (MDR)-reversing activity	[78]
**111,112**	Antiproliferative activity	[79]
**114**	Inhibitory effects on nitric oxide production	[81]
**115**	Used to alleviate abdominal pain and treat cancer	[82]
**118,119**	Immunosuppressive activities	[85]
**120–122**	Strong cytotoxicity and antibacterial activity	[86]
**123**	Antibacterial activity	[75]
**137,138**	Moderate cytotoxicity against various cancer cell lines	[97]
**142**	Cytotoxic activity against BGC-823 and HepG2 cells	[100]
**170**	Moderate cytotoxicity against five human cancer cell lines	[110,111]

**Table 2:** Summarized biological activity of B-norsteroids.

Steroid No.	Reported Activity of B-Norsteroids	Ref.
**189**	Strong cytotoxic effects against human leukemia HL-60 cells	[130,131]
**205–207**	Strong cytotoxicity against the P388 mouse leukemia cell line	[146]
**219**	Anti-inflammatory and ichthyotoxic properties	[149,150,151,152]
**221,222**	Antimicrobial activity	[155]
**242**	Cytotoxic effects against MCF-7	[166]
**243**	Inhibitory activity against 11β-HSD1	[167]
**251**	Antifungal against human pathogens	[170]
**252**	Cytotoxicity against human cancer cell lines	[171]
**260**	Cytotoxicity against leukemia cells	[178]
**264**	Cytotoxicity against various cancer cell lines	[181]
**275**	Antibacterial activity	[189]
**284**	Strong cytotoxic activities against several cancer cell lines	[197]
**286,287**	Antihypercholesterolemic and hypolipidemic activities	[199]

**Table 3:** Summarized biological activity of C-norsteroids.

Steroid No.	Reported Activity of C-Norsteroids	Ref.
**288–290**	Cytotoxic activities toward various cancer cell lines	[204]
**291**	Cytotoxicity towards breast cancer cells	[205]
**294**	Cytotoxicity towards lung and bone cancer cells An inhibitory effect on (h)p300	[206,207]
**305**	Antitrypanosomal and antiplasmodial effects	[216]
**306**	Inhibition of Na+, K+-ATPase	[217]
**307**	Antibacterial activity	[218]
**309**	Inhibitory activity against influenza A	[219]
**310**	Anti-inflammatory activity	[220]
**319**	Cytotoxicity against human cancer cell lines	[223]
**320–324**	Cytotoxic activities against various human cell lines	[224,225]
**352**	Antiproliferative effects	[234]
**363,364**	NO inhibitory activity	[238]
**385–387**	Cytotoxicity against various human tumor cell lines	[251]
**390**	Anticoagulant activity	[252]
**394**	Antifeedant activity	[255]
**406,407**	Antiplatelet aggregation activity	[259]
**408–416**	Strong cytotoxicity against various cancer cell lines	[260]

**Table 4:** Summarized biological activity of D-norsteroids.

Steroid No.	Reported Activity of D-Norsteroids	Ref.
**423–432**	Reproductive system conditions, hypertension, and anticancer activity	[265,266,267,268,269,270,271,272,273,274]
**435**	Insecticidal activity	[277,278]
**436–438**	Antiausterity agents, cytotoxicity against the PANC-1 human pancreatic cancer cell line	[279]
**441,442**	Anticancer activity	[282]
**453–455**	Veterinary anticancer drugs	[300]
**474**	Antimicrobial activity	[313]
**486–489**	Antimicrobial activity	[317]

**Table 5:** Summarized biological activity of meroterpenoids.

No.	Reported Activity of Meroterpenoids	Ref.
**496**	Protein kinase inhibitor	[328]
**497,498**	Strong ichthyotoxic and antifeedant activity	[329]
**524**	Strong cytotoxicity against L1210 and KB cancer cells	[338]
**526–528**	Cytotoxic activity against P388 cell lines	[340,341]
**544**	Cytotoxic activity against the PC3 and PBL-2H3 cell lines	[345]
**556**	Antifungal activity	[349]
**561**	Antimycobacterial activity	[351]
**567–572**	Antibacterial activity	[354]
